# Nematic bits and universal logic gates

**DOI:** 10.1126/sciadv.abp8371

**Published:** 2022-08-19

**Authors:** Žiga Kos, Jörn Dunkel

**Affiliations:** ^1^ Department of Mathematics, Massachusetts Institute of Technology, 77 Massachusetts Avenue, Cambridge, MA 02139, USA.; ^2^ Faculty of Mathematics and Physics, University of Ljubljana, Jadranska 19, 1000 Ljubljana, Slovenia.; ^3^ Jožef Stefan Institute, Jamova 39, 1000 Ljubljana, Slovenia.

## Abstract

Liquid crystals (LCs) can host robust topological defect structures that essentially determine their optical and elastic properties. Although recent experimental progress enables precise control over nematic LC defects, their practical potential for information storage and processing has yet to be explored. Here, we introduce the concept of nematic bits (nbits) by exploiting a quaternionic mapping from LC defects to the Poincaré-Bloch sphere. Through theory and simulations, we demonstrate how single-nbit operations can be implemented using electric fields, to construct LC analogs of Pauli, Hadamard, and other elementary logic gates. Using nematoelastic interactions, we show how four-nbit configurations can realize universal classical NOR and NAND gates. Last, we demonstrate the implementation of generalized logical functions that take values on the Poincaré-Bloch sphere. These results open a route toward the implementation of classical digital and nonclassical continuous computation strategies in topological soft matter systems.

## Teaser

Topological defects can be used as information bits to perform computations and universal logic operations in liquid crystals.

## INTRODUCTION

Bits are the fundamental units of binary digital computation and information storage. Similar to an idealized universal Turing machine (*1*), classical digital computers represent bits as two discrete voltage states, commonly labeled 0 and 1. Accordingly, electronic digital circuits process information by manipulating deterministic bit sequences · · 01011 · · with the help of logic gates. Notwithstanding the historical success of classical bit-based computation, it has long been suggested that some practically relevant problems could be solved by performing parallel computations in larger or nondiscrete state spaces (*2*–*7*). A popular example is quantum computers (*8*), which promise substantially faster search (*9*) and factoring (*10*) algorithms. In parallel, several other promising information processing strategies in classical systems are currently also being explored, including DNA-based computation (*11*), analog-computing in cells (*12*), chemical computers (*13*, *14*), microfluidic (*15*–*18*) and mechanical (*19*) digital logic, or holonomic computation (*20*) in non-Abelian mechanical (*21*) systems. Independent of whether such nonstandard approaches will eventually result in scalable computing technologies, their exploration has generally led to a better experimental and theoretical understanding of the underlying physical, chemical, and biological systems.

**Fig. 1.**
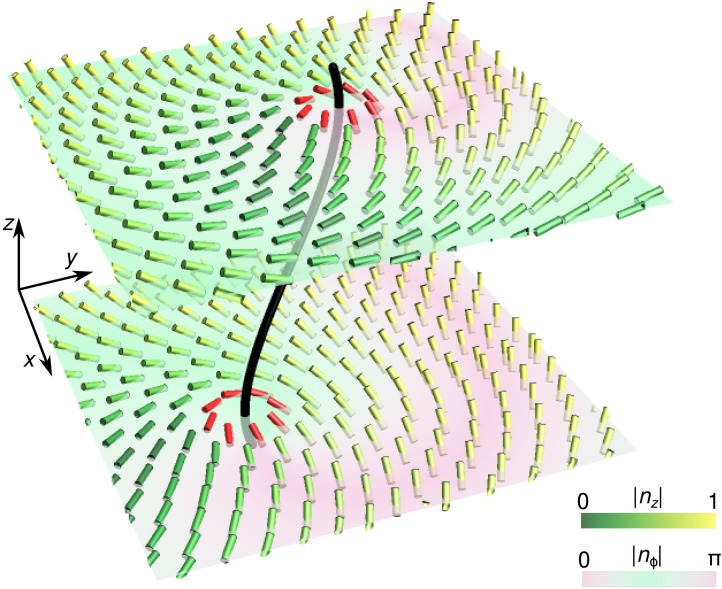
Nbits pinned to an LC defect line. The local nematic director field ***n***(***r***), indicated by cylindrical bars, rotates by π along closed curves encircling the defect line (black). The director field is colored by its out-of-plane component, *n_z_*(***r***), while *xy*-planes are colored by the director’s azimuthal orientation *n*_ϕ_(***r***) relative to the *x* axis. The near-field director profile (red) close to the defect line defines the nbit state (see also fig. S1). The vertical direction may be interpreted as either a spatial or a time dimension.

A widely studied class (*22*–*24*) of continuous soft matter systems that can be accurately controlled experimentally (*25*, *26*) but whose computational potential has not yet been systematically investigated are nematic liquid crystals (LCs). Composed of rod-like molecules, LCs can host topological defects that are structurally robust against external perturbations, yet can be precisely manipulated through boundary conditions (*25*) and electric fields (*26*), as well as locally reconfigured with lasers (*27*). Building on recent theoretical work (*28*) that identified a direct relation between string-like LC defects and quaternions, we demonstrate here that such topological defects can be used as both classical binary and nonclassical continuous nematic bits (nbits; Fig. 1). By deriving a reduced dynamical description from electro-nematic LC theory (*29*, *30*), we show how individual nbits, which correspond to points on the Poincaré-Bloch sphere (*31*), can be transformed in analogy with Pauli, Hadamard, and other typical single-qubit gates (*32*, *33*) using electric fields. Generalizing to multi-nbit states, we find that nematoelastic interactions can cause strong correlations in an ensemble of nbit pairs, suggesting that such interactions can be used to realize logic functions. We confirm this prediction by demonstrating universal classical NAND and NOR gates as well as generalized continuous logic functions in simulations for experimentally feasible nematic LC parameters. Our numerical results, combined with quantitative estimates of the typical energetic costs and time scales associated with physical nbit-manipulations, suggest that nbit-circuits can be implemented with existing LC technology.

## RESULTS

### Defining and transforming single nbit states

**Fig. 2.**
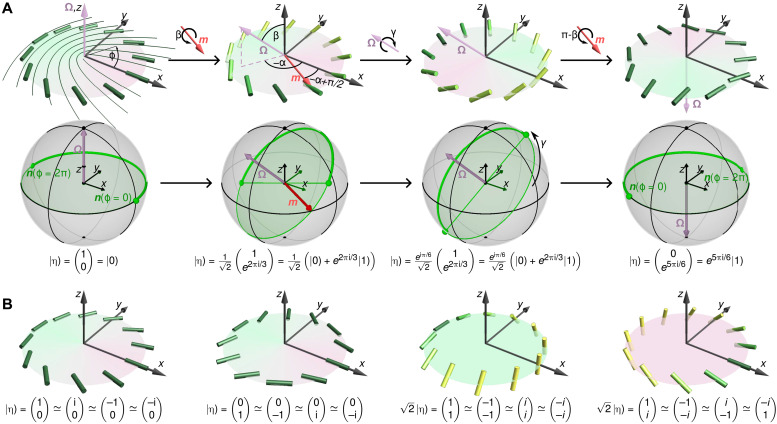
Single-nbit transformations. (**A**) The director profile ***n***_0_(***r***), describing a +1/2-defect aligned with the positive *x* direction, defines the nbit state ∣0). Arbitrary single-nbit states ∣η) are obtained from ∣0) through simultaneous local director rotations, corresponding to near-field profiles ***n***(***r***) = η***n***_0_(***r***)η^†^ with η given by Eq. (1); see also movies S1 and S2. Each nbit ∣η) has a distinguished normal axis λ**Ω**, λ ∈ ℝ, spanned by an axial unit vector **Ω** perpendicular to the director field, **Ω** · ***n***(***r***) = 0; we choose **Ω** to point along the positive *z* direction for ∣0). Orthogonal states, such as ∣0) and ∣1), have antiparallel **Ω** vectors. Top: Example sequence showing how nbit ∣0) can be continuously transformed to the nbit *e*^*i*5π/6^ ∣1) corresponding to a −1/2-defect profile. To this end, ∣0) is first homogeneously rotated around the axis ***m*** = (cos (α + π/2), sin (α + π/2),0) by an angle β, yielding an intermediate nbit having a rotated normal **Ω** with spherical polar coordinates (α, β). Subsequent director rotation around **Ω** = (α, β) by an angle γ results in the arbitrary single-nbit state ∣η) = ∣α, β, γ). In the depicted example sequence, the second intermediate state is ∣η) = ∣− 2π/3, π/4, π/3). Performing an additional rotation around ***m*** by π − β leads to the −1/2-defect nbit *e*^*i*5π/6^ ∣1); for comparison, the nbit ∣1) corresponds to a −1/2-defect aligned with the *x* axis. Bottom: On the nematic order-parameter unit sphere, an nbit director field ***n*** traces out a great semi-circle (thick green curves) from ***n***(ϕ = 0) to ***n***(ϕ = 2π), which lies in a plane perpendicular to **Ω**. (**B**) Four distinct nbits with **Ω** pointing in the direction of *z*, −*z*, *x*, and −*y*, respectively. The symbol **≃** indicates nbit states with identical near-field director profile but different global topology (see fig. S2).

Nematic LCs are assemblies of aligned rod-like molecules. By imposing suitable boundary anchoring conditions (*25*, *27*) or external electric fields (*26*), the molecules’ global orientational order can be locally broken at singular defect points in two-dimensional (2D) or lines in 3D (Fig. 1 and fig. S1) (*34*, *35*). Such topological defects (*24*, *36*) present particularly promising candidates for robust information storage as their existence is protected against thermal and other perturbations. To define an nbit, we consider an elementary +1/2 defect in the (*z* = 0)-plane of a 3D nematic LC, as shown in Fig. 2A (top/left). In this configuration, the defect center line passes through the coordinate origin, all the molecule directors lie on the same plane (with normal **Ω** along the *z* axis), and the symmetry axis of the texture is aligned with the *x* axis. We refer to this configuration as reference nbit state ∣0) throughout and denote the associated director profile as ***n***_0_(***r***), where ***r*** is the in-plane distance vector from defect line (fig. S1). Keeping the defect location fixed, arbitrary nbit states ∣η) correspond to rotated director profiles ***n***(***r***), obtained from ∣0) by turning each director ***n***_0_(***r***) individually by the same angle θ about a common axis spanned by the unit vector ***a*** = (*a_x_*, *a_y_*, *a_z_*). To highlight the mathematical parallels between nbits and qubits, we express these local director rotations using quaternions (*28*, *37*)

$$\eta =1\;\text{cos}\;\frac{\theta }{2}+i(a_{x}\sigma_{x}+a_{y}\sigma_{y}+a_{z}\sigma_{z})\text{sin}\;\frac{\theta }{2}$$ (1)

where σ*_x_*, σ*_y_*, and σ*_z_* correspond to the Pauli matrices. The director profiles of the nbits ∣η) and ∣0) are then linked by quaternion product ***n***(***r***) = η ***n***_0_(***r***) η^†^, where the director is written in terms of Pauli matrices, ***n*** = *n_x_*σ*_x_* + *n_y_*σ*_y_* + *n_z_*σ*_z_*. Because each quaternion of the form Eq. 1 has an SU(2) matrix representation $\left(\begin{array}{cc} c_{1} & -{\bar{c}}_{2}\\ c_{2} & {\bar{c}}_{1} \end{array}\right)$ with ∣*c*_1_∣^2^ + ∣*c*_2_∣^2^ = 1, we can identify ∣η) with the first column of the SU(2) matrix corresponding to η.

**Fig. 3.**
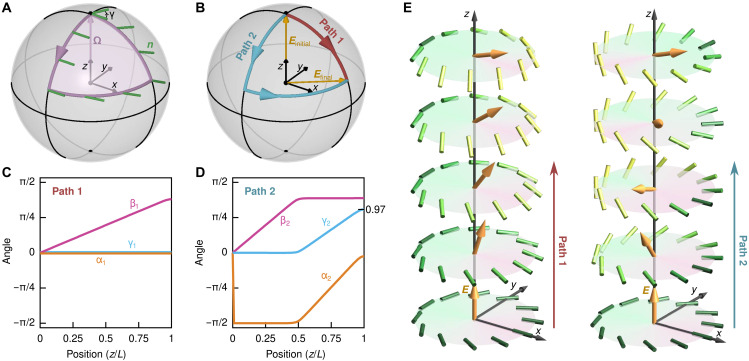
Transforming nbits with electric fields. An initial nbit state in the plane *z* = 0 can be transformed into an arbitrary target nbit at *z* = *L* by applying a suitable electric field protocol ***E***(*z*) that smoothly adjusts the orientation of the director normal vector **Ω** along *z* and realizes the global phase angle γ in Eq. 4 by choosing an appropriate electric field path. (**A**) The nbit phase γ corresponds to a Berry phase and can be changed by moving **Ω** around a closed path. By parallel transport, the change in γ is equal to the area enclosed by the path. (**B**) Two example protocols ***E***(*z*), corresponding to distinct paths 1 and 2, used in simulations of Eq. 5. Both paths go from the same initial field ***E***_initial_(*z* = 0) to the same final field ***E***_final_(*z* = *L*) but result in a different final phase γ. (**C** and **D**) Angles α, β, and γ, measured along *z* in simulations of Eq. 5 for the two protocols ***E***(*z*) from (B), starting with reference nbit ∣0) at *z* = 0. (C) For path 1, α_1_ rapidly jumps to its stationary value, while β_1_ grows linearly with *z* and γ_1_ remains constant. (D) For path 2, α_2_ and β_2_ approach similar final values as for path 1, but γ_2_ reaches a substantially larger final value close to the area of π/3 enclosed by both paths. (**E**) Simulated director configurations for the two electric field protocols from (B) to (D). Both protocols lead to final nbit states having the same normal vector **Ω**, which is determined only by the final electric field orientation at *z* = *L*. However, the final director profiles differ through a γ_2_ rotation around **Ω**. Any final nbit state ∣η) can be realized using this strategy.

To obtain a geometrically intuitive, equivalent representation of the spinor ∣η), it is convenient to decompose rotations of the reference nbit ∣0) into two steps (Fig. 2A): First, the director field ***n***_0_ of ∣0) is rotated around the axis ***m*** = (cos (α + π/2), sin (α + π/2),0) by an angle β, yielding an intermediate nbit having a rotated normal **Ω** with spherical polar coordinates (α, β); thereafter, the directors are rotated around the new normal **Ω** by an angle γ. This gives the following geometric representation of an nbit

$$|\eta )=|\alpha ,\beta ,\gamma )=e^{i\frac{\gamma }{2}}(\begin{array}{c} \text{cos}\;\frac{\beta }{2}\\ e^{-i\alpha }\text{sin}\;\frac{\beta }{2} \end{array})$$ (2)

where the global phase γ corresponds to a Berry phase (Fig. 3A). As evident from Eq. 2, nbits for which the global phase γ differs by π, 2π, or 3π exhibit the same local director profile in the vicinity of the defect line (Fig. 2B). However, such locally equivalent configurations can be distinguished by their global topology, as demonstrated in movies S1 and S2 and fig. S2.

Moreover, director rotations in Eq. 2 make explicit that the two basis states

$$|0)=(\begin{array}{c} 1\\ 0 \end{array}),\;\;|1)=(\begin{array}{c} 0\\ 1 \end{array})$$ (3)

correspond to *x* axis–aligned +1/2 and −1/2 defects, respectively (Fig. 2B). Thus, an arbitrary nbit ∣η) can be expressed as a normalized complex linear combination of these two elementary defect states

$$|\eta )=c_{1}|0)+c_{2}|1)=e^{i\frac{\gamma }{2}}[\text{cos}\;\frac{\beta }{2}|0)+e^{-i\alpha }\text{sin}\;\frac{\beta }{2}|1)]$$ (4)

with the complex vector superposition encoding physical director rotations. In particular, the above construction shows that single-nbit states are mathematically equivalent to single-qubit states. It should be stressed, however, that despite sharing a mathematically equivalent state space, nbits and qubits are not physically equivalent. In particular, classical nbits are governed by a dissipative dynamics, whereas qubits obey wave-like Schrödinger-Pauli dynamics. As a consequence, multi-qubit states can produce interference phenomena that are unlikely to find direct counterparts in nbit-systems, implying differences in their computational capabilities. Notwithstanding such differences, we will see below that nbits can be used to implement both classical binary logic functions and generalized continuous logic functions.

### Controlling nbits with electric fields

The ability to access and manipulate individual nbits is essential for the experimental implementation of nematic LC computers. To demonstrate a practically feasible protocol, we generalize recent advances in the electro-nematoelastic transformation of LC defects (*29*, *30*). Starting from the field equations of LC hydrodynamics, one can show (Supplementary Materials) that the adiabatic relaxation dynamics of an nbit-quarternion Eq. 1 in a slowly varying, sufficiently strong electric field ***E*** = *E_x_*σ*_x_* + *E_y_*σ*_y_* + *E_z_*σ*_z_* is determined by the matrix evolution equation

$$\frac{d\eta }{\mathrm{dt}}=\frac{K}{\Gamma }\frac{d^{2}\eta }{dz^{2}}-\frac{\epsilon_{a}}{4\Gamma }\text{Tr}(\eta \sigma_{z}\eta^{†}E)E\eta \sigma_{z}-\lambda \eta$$ (5)

where *K* is the elastic constant of the nematic director field, Γ denotes the rotational diffusion constant, and ϵ_a_ is the dielectric anisotropy. The Lagrange multiplier λ preserves the SU(2) structure of η. In the remainder, we shall focus on LCs with negative dielectric anisotropy, ϵ_a_ < 0, such as *N*-(4-Methoxybenzylidene)-4-butylaniline (MBBA) (*38*); analogous control strategies can be devised for materials with ϵ_a_ > 0.

For LCs with ϵ_a_ < 0, Eq. 5 implies that the director normal axis **Ω** of an nbit prefers to align with the electric field ***E***. This means that one can program the polar coordinates (α, β) of ∣η) through the instantaneous direction of ***E***. In addition, the global Berry phase γ of an nbit can be set by moving **Ω** around a closed path (Fig. 3A). Suitably designed spatial (or temporal) electric field protocols can thus be used to transform an initial nbit into any desired target nbit (Fig. 3). Figure 3 (B to E) shows two example protocols ***E***(*z*) that transform nbit ∣0), which is localized in the plane *z* = 0, into distinct target nbits at *z* = *L*. The two protocols connect the same initial and final field values, ***E***(*z* = 0) and ***E***(*z* = *L*), via two different paths 1 and 2 (Fig. 3B), so that the final nbit states have similar normal coordinates (α, β) but differ in their global phase angles γ (Fig. 3, C and D). The corresponding nbit director fields, computed numerically (Materials and Methods) from the stationary solutions of Eq. 5 along both paths, can be seen in Fig. 3E. The same electric control strategy can be used to implement nematic logic gates.

### Nematic logic gates and projective measurements

**Fig. 4.**
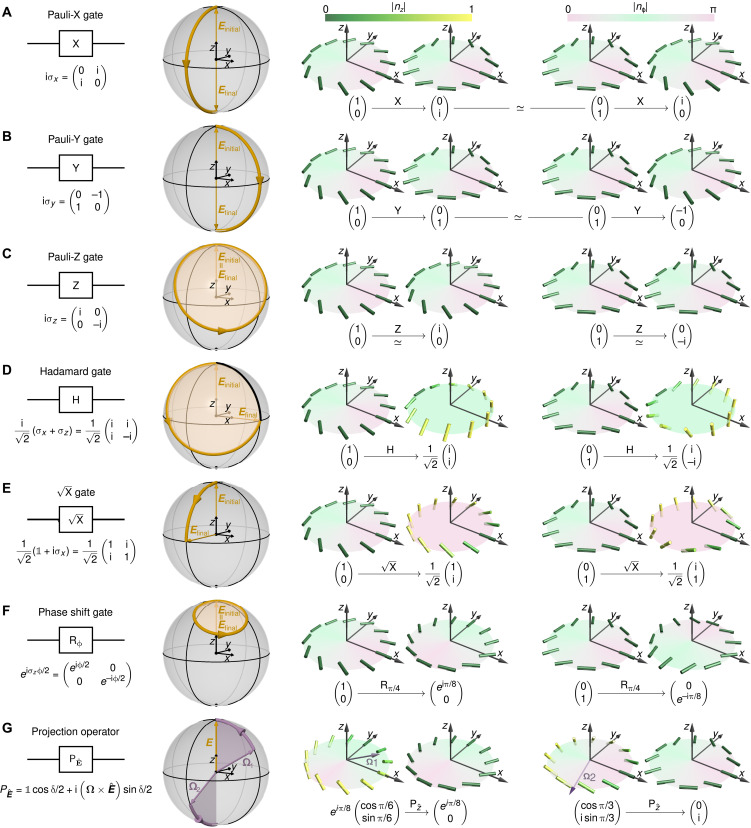
Single-nbit logic gates and projection operator. Typical quantum logic gates (first column) acting on single-nbit states can be realized by rotating an applied electric field. The second column shows the electric field path required to realize logic operations on the ∣0) bit. Columns 3 and 4 depict initial and final director configurations for logic gates applied to ∣0) or ∣1) nbits, respectively. (**A** to **C**) Nematic Pauli gates perform a rotation by π around the *x*, *y*, or *z* axis, respectively. Pauli-X and Pauli-Y gates transform the two basis states ∣0) and ∣1) into each other (up to a global phase). The Pauli-Z gate adds a phase π/2 to ∣0) and a phase **−**π/2 to ∣1). (**D**) The Hadamard gate rotates an nbit by π around the $\hat{x}+\hat{z}$ axis, transforming ∣0) and ∣1) into tangential twist defect profiles. (**E**) The $\sqrt{X}$ gate performs a π/2 rotation around the *x* axis, transforming ∣0) and ∣1) into radial twist defect profiles. (**F**) The phase shift gate $\sqrt{R_{\phi }}$ induces a rotation by ϕ around the *z* axis, preserving the angle β of an nbit. The action of $\sqrt{R_{\phi }}$ on the director fields of ∣0) and ∣1) is equivalent to a rigid rotation around the *z* axis by 2ϕ and 2ϕ/3, respectively. (**G**) $P_{\hat{E}}$ projects the director normal **Ω** onto the axis of a sufficiently strong (Supplementary Materials) electric field $\hat{E}$ by rotating the nbit around **Ω × *E***, where the rotation angle $\delta =\text{sign}(\Omega \cdot \hat{E})\text{acos}(\text{sign}(\Omega \cdot \hat{E})\Omega \cdot \hat{E})$ corresponds to the angle between **Ω** and the electric field axis. For a projective measurement along the *z* direction, nbits with **Ω** pointing into the northern or southern hemispheres become projected onto the ∣0) or ∣1) subspaces, respectively.

Because, according to Eq. 4, single-nbit states ∣η) are equivalent to elementary qubits, one can implement direct nematic analogs of many standard quantum logic gates (*39*) by applying suitable electric field profiles. Figure 4 summarizes realizations of commonly used gates, showing the electric field protocols required to transform ∣0) and also the final nbit states when logic operations are performed on ∣0) or ∣1), respectively. Specifically, we demonstrate the actions of the three nematic Pauli gates (Fig. 4, A to C), the Hadamard gate (Fig. 4D), $\sqrt{X}$ gate (Fig. 4E), and the phase shift gate (Fig. 4F). As a general practical rule, the initial electric field of the gate needs to be aligned with the normal axis **Ω** of the nbit ∣η) to which the gate operation is applied. Experimentally, the determination of **Ω** can be done optically and noninvasively in LC materials (*40*), in contrast to quantum systems. Another interesting class of operations, corresponding to projective measurements, can be realized by applying electric fields with a fixed direction. For example, by fixing the field vector ***E*** in Eq. 5 parallel to the *z* direction, all nbits with **Ω** pointing into the norther hemisphere become projected onto the ∣0) subspace, whereas all nbits with **Ω** in the southern hemisphere become projected onto the ∣1) subspace (Fig. 4G). These single-nbit operations provide a basis for implementing computations in nematic LC systems.

### Two-nbit states

**Fig. 5.**
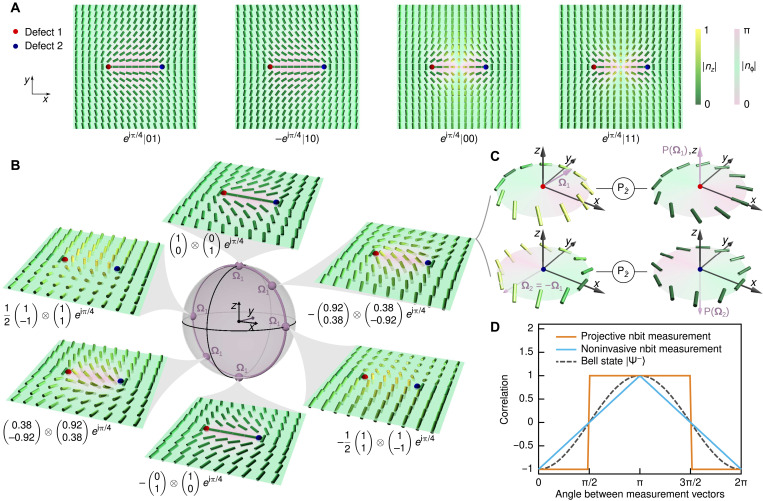
Two-nbit states and Bell-type correlations. A two-nbit product state ∣η_1_η_2_) = ∣η_1_) ⊗ ∣η_2_) is a topological defect pair, with the local director field structure around the defects 1 and 2 defining the single-nbits ∣η_1_) and ∣η_2_), respectively. (**A**) The two-nbit states *e*^*i*π/4^ ∣01) and −*e*^*i*π/4^ ∣01) are free-energy minima, whereas *e*^*i*π/4^ ∣00) and *e*^*i*π/4^ ∣11) exhibit an umbilic soliton (*35*) at the center. (**B**) Representative examples of energetically equivalent (fig. S5) two-nbit product states with the director far-field parallel to the *y* direction, selected from the ensemble manifold Ψ^**−**^ of antiparallel nbit states with **Ω**_1_ = **−Ω**_2_. The two-nbit states in the Ψ^**−**^ ensemble can be strongly correlated under projective measurements due to nematoelastic interactions. (**C**) A global projective measurement, performed by applying a *z*-aligned electric field, $\hat{E}\|\hat{z}$, that projects both **Ω**_1_ and **Ω**_2_ along the *z* direction (Fig. 4G), collapses each two-nbit state in the Ψ^**−**^ ensemble to a ±1/2 defect pair. (**D**) The choice of the local measurement procedure determines the observed nbit correlations, defined as the ensemble average ${\langle \text{sign}(\Omega_{1}\cdot {\hat{v}}_{1})\cdot \text{sign}(\Omega_{2}\cdot {\hat{v}}_{2})\rangle }_{\Psi^{-}}$ where ${\hat{v}}_{1}$ and ${\hat{v}}_{2}$ are the local measurement vectors applied to nbit 1 and 2, respectively. Noninvasive measurements produce the classically expected piecewise linear correlation dependence on the relative angle between ${\hat{v}}_{1}$ and ${\hat{v}}_{2}$ (blue). By contrast, projective measurements performed by successively applying local electric fields with orientations ${\hat{E}}_{1}={\hat{v}}_{1}$ and ${\hat{E}}_{2}={\hat{v}}_{2}$ to the nbits can result in nearly perfect correlation or anticorrelation (orange), provided the time delay between the two measurements is sufficiently long to allow for nematoelastic relaxation.

The above framework can be generalized to systems with two and more interacting nbits. To construct their mathematical description, we consider configurations of two nearby defects in the *xy*-plane (Fig. 5A and figs. S3 and S4). The defects represent nbits ∣η_1_) and ∣η_2_), respectively, and we can describe their joint state by the tensor product ∣η_1_) ⊗ ∣η_2_). Analogous to the single-nbit case above, a (+1/2, −1/2) defect pair aligned with the *x* axis defines the reference state *e*^*i*π/4^ ∣0) ⊗ ∣1) ≡ *e*^*i*π/4^ ∣01), where the global phase factor reflects the rotation of the −1/2 defect (Fig. 5A, left) with respect to its orientation in Fig. 3B. Arbitrary two-nbit product states can be realized by rotating the local director fields around each defect, with the individual phases γ_1_ and γ_2_ determining the global two-nbit phase γ = γ_1_ + γ_2_. For example, by rotating the directors of each of the two nbits in the reference state *e*^*i*π/4^ ∣0) ⊗ ∣1) by an angle π around the *y* axis, corresponding to the action of the Pauli-Y gate (Fig. 4B), one obtains the state −*e*^*i*π/4^ ∣1) ⊗ ∣0) ≡ − *e*^*i*π/4^ ∣10) shown in the second panel of Fig. 5A. General nematic product states are given by *e*^*i*γ*_ij_*^ ∣*i*) ⊗ ∣*j*), corresponding to pairs of suitably rotated topological defects (Fig. 5, A and B). Ensembles of two-nbit states can exhibit strong statistical correlations, facilitated by nematoelastic interactions of the director fields.

### Nematoelastic interactions

Nematoelastic interactions may provide a resource for nematic computation. For example, when the defects 1 and 2 forming a two-nbit state are embedded into a homogeneous director far-field, their energetic equilibrium configurations correspond to director profiles in which the local normal vectors **Ω**_1_ and **Ω**_2_ are perfectly antialigned, **Ω**_1_ = −**Ω**_2_ (Fig. 5B). Moreover, the common axis of **Ω**_1_ and **Ω**_2_ can point in an arbitrary direction, defining an ensemble Ψ^−^ of energetically degenerate states if the nematic material can be appropriately described by a single dominant elastic constant *K* (Fig. 5B). This leads to a strong nematoelastic coupling: If one nbit is slowly rotated, the other nbit will adiabatically follow to ensure that **Ω**_1_ = −**Ω**_2_. Similarly, by enforcing an umbilic soliton (*35*) in the system (Fig. 5A), one can realize an ensemble Φ^+^ of two-nbit states with **Ω**_1_ = **Ω**_2_ (fig. S6). The nonlocal nematoelastic coupling of the nbit orientations can lead to Bell-type correlations when projective measurements are performed on individual nbits (Fig. 5, C and D). As we will show, such coupling has a well-defined elastic time scale. From a practical perspective, this suggests that nematoelastic interactions can be exploited as a computational resource.

### Noninvasive versus projective nbit measurements

State-of-the-art optical techniques (*40*) can measure and distinguish the defect textures in LC materials without perturbing them, whereas applied electric fields can be used to reorient LCs (*26*) along a preferred axis (Figs. 4G and 5C). This means that nbit systems allow for both noninvasive and projective measurements. Depending on the details of the experimental measurement protocol and the characteristic time scale of the nematoelastic interactions, two-nbit ensembles can exhibit statistical correlations that are weaker or stronger than those of quantum Bell states. Such correlations can provide a computational resource for realizing logic operations in LCs. We demonstrate the dependence of the correlations on the measurement protocol for the Ψ^−^ ensemble, consisting of two-nbits states with **Ω**_2_ = −**Ω**_1_ where **Ω**_1_ is uniformly distributed on the 2D unit sphere (Fig. 5B). We compare the nematic correlation strength with the expected spin correlations for the maximally entangled two-qubit Bell-state (*39*, *41*) $|\Psi^{-}\rangle =(|0\rangle ⊗|1\rangle -|1\rangle ⊗|0\rangle )/\sqrt{2}$ in a standard projective quantum measurement. If the state of the first qubit in ∣Ψ^−^〉 is measured along some direction ${\hat{v}}_{1}$ and that of the second qubit along ${\hat{v}}_{2}$, then the observed correlations are known to be stronger than for optimized classical “local realist” (*39*) imitations (Fig. 5D). In the case of a noninvasive nbit measurement, the analytically calculated ensemble-averaged correlation function ${\langle \text{sign}(\Omega_{1}\cdot {\hat{v}}_{1})\cdot \text{sign}(\Omega_{2}\cdot {\hat{v}}_{2})\rangle }_{\Psi^{-}}$ exhibits the classically expected linear dependence on the angle between ${\hat{v}}_{1}$ and ${\hat{v}}_{2}$ (blue curve in Fig. 5D). By contrast, projective nbit measurements can lead to substantially stronger correlations due to the nematoelastic coupling between the nbits. Applying a global electric field along the *z* axis projects the nbits of a Ψ^−^ configuration onto a pair of ±1/2 defects, corresponding to states locally equivalent to *e*^*i*π/4^∣01) or −*e*^*i*π/4^∣10) (Fig. 5C). Similarly, a global projection along *x* transforms Ψ^−^ configurations into states that are proportional to tensor products of $|+)\equiv [|0)+|1)]/\sqrt{2}$ and $|-)\equiv [|0)-|1)]/\sqrt{2}$. Particularly interesting correlations due to nematoelastic interactions arise when two local projective measurements along different axes are performed successively on the two nbits. To see this, let us assume that nbit 1 of a Ψ^−^ configuration is first projected along ${\hat{v}}_{1}$ by a local electric field with orientation ${\hat{E}}_{1}={\hat{v}}_{1}$. After the measurement, **Ω**_1_ will point along $+{\hat{v}}_{1}$ or $-{\hat{v}}_{1}$ and, given sufficient time, the nematoelastic interactions will reorient the axis of the second nbit until **Ω**_2_ = −**Ω**_**1**_ is reached. Thus, subjecting the reoriented nbit 2 to a second projective measurement along ${\hat{E}}_{2}={\hat{v}}_{2}$ yields the strongest possible correlation (orange curve in Fig. 5D). In practice, one can tune the correlation strength in an nbit system by adjusting the time delay between the projection measurements relative to the nematoelastic relaxation time scale.

### Multi-nbit logic operations

**Fig. 6.**
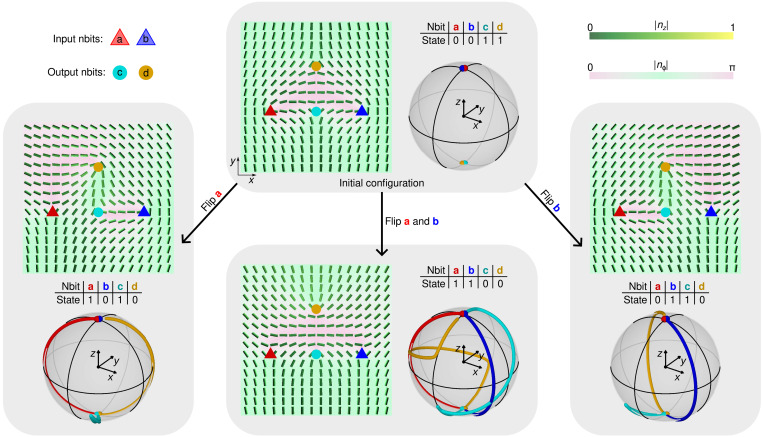
Universal classical logic gates. In the initial configuration (**top**), the two input nbits ‘a’ and ‘b’ are in the 0 state [corresponding to ∣0)] and two output nbits ‘c’ and ‘d’ are in the 1 state [corresponding to ∣1)]. The initial configuration is transformed by changing the director field in the vicinity of the input nbits and observing the nematoelastic response of the output nbits; the Poincaré-Bloch spheres show the evolution of the nbit states during each reconfiguration process. (**Left**) nbit ‘a’ is flipped into the 1 state by rotating its local director field around the (cos (−3π/8), sin (−3π/8),0) axis, causing the output nbit ‘d’ to change from 1 to 0 while leaving ‘b’ and ‘c’ unchanged (up to a phase factor). (**Right**) The director of input nbit ‘b’ is rotated around the (cos (−π/8), sin (−π/8),0) axis, causing a flip in the state of the output nbit ‘d’. (**Bottom-middle**) Simultaneous flipping of the two input nbits ‘a’ and ‘b’ results in a 0 state for both output nbits. Thus, nbits ‘c’ and ‘d’ obey classical NAND and NOR gate transformation rules, respectively. Topologically, gate operations involving multiple nbits create either nonisolated singularities in the projection of the director field on the plane or additional umbilic solitons (fig. S6), thus preserving the Poincaré-Hopf theorem when switching between nbit states. The nbits are pinned and cannot move or mutually annihilate.

Using strong correlations between nbits, we can perform classical logic operations in systems of multiple nbits. Figure 6 and movie S3 show a logical operation on a system of four nbits, where, in the initial configuration, two input nbits ‘a’ and ‘b’ are in a ∣0) state (up to a phase factor), and two output nbits ‘c’ and ‘d’ are in a ∣1) state. One or both input nbits are then flipped into the ∣1) state, tracking the output response. After the equilibrium is reached, output nbits are observed to be in either the ∣0) or ∣1) state. The combined truth table reveals that the nbit transformations in Fig. 6 realize a universal classical NAND gate for output nbit ‘c’ and a universal NORgate for output nbit ‘d’. The NAND and NOR responses of the output nbits are achieved by an explicit choice of the nbit positions and the axis of rotation. For other geometries, different responses may be measured that could also go beyond the discrete ∣0) or ∣1) states.

**Fig. 7.**
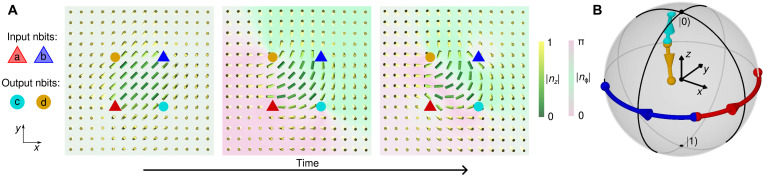
Multi-nbit operations beyond classical logic functions. By varying the defect positions and initial multi-nbit configuration, it is possible to implement generalized continuous logical operations on a Poincaré-Bloch sphere, where input and output nbits are not limited to switching between ∣0) and ∣1) but can assume any superposition of these states. The figure shows an example, in which defects are arranged in a square pattern and input nbits ‘a’ and ‘b’ are transformed from ∣*a*)_init_,∣ *b*)_init_ ∝∣0) + *e*^iπ/4^ ∣1) to ∣*a*) ∝∣0) + *e*^−iπ/4^∣1) and ∣*b*) ∝ ∣0) + *e*^i3π/4^ ∣1), respectively (prefactors are omitted in the notation). Output nbits ‘c’ and ‘d’ respond to the input configuration, transforming from ∣*c*)_init_,∣*d*)_init_ ∝ ∣0) + *e*^−i3π/4^ ∣1) to ∣*c*) ∝ 0.87 ∣0) + 0.50*e*^−2.32i^ ∣1) and ∣*d*) ∝ 0.50 ∣0) + 0.87*e*^−2.32i^ ∣1), respectively. (**A**) Timeline of input and output nbit transformations. (**B**) Paths of nbit transformations traced on a Poincaré-Bloch sphere.

A general multi-nbit logic operation does not involve only discrete 0 and 1 bits but is, in principle, determined by a mapping from Poincaré-Bloch spheres of the input states to the Poincaré-Bloch sphere of the output. We show an example of such a nondigital operation in Fig. 7, where input and output nbits are always in a superposition of ∣0) and ∣1) states. For the chosen spatial arrangement and initial configuration of the nbits, transforming the input nbits along the equator of the Poincaré-Bloch sphere results in a change of the polar angle on the Poincaré-Bloch sphere of the output nbits.

## DISCUSSION

### Time scales and energetic costs

The rate at which logic gate operations on nbits can be performed depends on both the elastic relaxation time scale of the LC material, τ_elastic_ ≃ Γ*R*^2^/*K*, where *R* is the characteristic cylindrical radius of the nbit line-defect domain, and the material’s response time in an applied electric field, τ_electric_ ≃ Γ/(∣ϵ*_a_*∣*E*^2^). Elementary nbit transformations require τ_electric_ ≪ τ_elastic_ to realize nondirect paths between the initial and final nbit direction (Fig. 3, A and B). This condition is satisfied for commonly used LC materials; for example, considering MBBA (*38*, *42*) with Γ = 0.076 Pa s, *R* = 10 μm, *K* = 5 *tpN*, ϵ_a_ = −0.7ϵ_0_ where ϵ_0_ is the vacuum permittivity, and *E* = 0.5 V/μm, one finds τ_elastic_ = 1.5 s and τ_electric_ = 0.049 s. To ensure that the director field can adiabatically follow the electric field path, the rate at which the electric control field is changed must be slower than the electric response rate 1/τ_electric_. At higher rates, backflow-coupling to the velocity field can affect logic gate operations, so electric fields should be manipulated using an optimized rate. Furthermore, for two-nbit measurements, the elastic relaxation scale separates the regimes of fast (noninvasive) measurements (τ_meas_ ≪ τ_elastic_) and slow projective measurements (τ_meas_ ≫ τ_elastic_); see Fig. 5D.

The energetic cost of manipulating an nbit can be estimated from the energy dissipation formula Eq. (S4). For example, transforming nbit ∣0) into ∣1) over a time period τ requires an energy of (Supplementary Materials)

$$E≃\frac{\pi^{3}\Gamma }{4\tau }LR^{2}$$ (6)

where *L* is the length of the defect line segment. Eq. 6 shows that fast reorientations require proportionally more energy. Similar to other physical bits, nbits can, in principle, also be affected by thermal perturbations. We estimate the typical time scale τ*_B_* to flip the nbit by considering ℰ ∼ *k_B_T*, where *k_B_* is the Boltzmann constant and *T* denotes temperature. Adopting MBBA parameters as above and assuming an nbit volume *LR*^2^ ∼ (10 μm)^3^ at room temperature, one finds τ*_B_* = 1.7 days, suggesting that nbits are thermally stable for typical operational time scales in the seconds range. Equation 6 implies that τ*_B_* can be increased by increasing the nbit volume, at the expense of requiring stronger electric fields for logical operations.

### Toward experimental implementation

The estimated characteristic time and energy scales suggest that commonly used LC materials can provide an experimental platform for realizing thermodynamically robust nbits. The theoretical framework developed above describes how specific computational operations can be performed through manipulation of individual or multiple nematic defects. Similar to biological (*12*), quantum (*8*), or chemical (*13*) computation systems, the practical implementation can be expected to provide challenges; recent numerical studies and experimental advances suggest that the nbit logic can be realized with currently available LC technology. In microfluidic chips, defect orientations and stability can be affected by the confining surfaces. To implement the full range of nbit operations, interactions between the nematic LC and boundaries ought to be minimized. A promising strategy is to embed the nematic phase between two isotropic boundary layers, as fully 3D simulations (*29*) showed a nematic sandwiched between two such boundary layers can be rotated to realize all possible orientations (*29*). Another feasible strategy is to use nematic materials and coatings with low surface anchoring (*43*). Such coatings can allow the director field in the nematic layer to turn to the vertical direction and thus enable the implementation of the above described gate operations.

Extending a previously validated theory framework (*27*, *44*), the above results show how nbits and universal logic gates can be controlled using local time-dependent electric fields. The creation of a single isolated nbit can be challenging due to its nonuniform far-field texture (Fig. 1); by contrast, the computationally more relevant multi-nbit states can be prepared with experimentally feasible uniform director far-fields (Figs. 5 to 7). In our simulations, the defects are pinned by imposing a lower local degree of order. In experiments, defect pinning can be implemented by impurities and local inclusions, which are known to localize defect positions (*45*, *46*). Pinning sites could also help to initialize nbit states during the phase transition from the isotropic to the nematic phase. Our present study focused on electric field–based protocols because structured electrodes have been successfully used to manipulate and control nematic director fields and defects in the past (*47*–*49*). Furthermore, recent experiments showed that nematic textures can be tuned with laser beams (*50*) and magnetic fields (*51*), suggesting a range of possible implementation strategies. LC interactions with light were used previously to implement optical logic gates (*52*–*54*). Future experimental realizations can also be aided by using materials with enhanced sensitivity to external fields, such as ferroelectric (*55*) or ferromagnetic (*56*) nematics.

To conclude, recent theoretical (*34*, *35*, *57*–*60*) and experimental (*25*–*27*, *61*, *62*) advances enable unprecedented control over topological structures in nematic LCs. Our above analysis provides a conceptual framework for storing and processing information in the textures of nematic fluids that, unlike hardwired solid-state devices, can be reconfigured and adapted. Similar to the development of quantum computation over the past three decades (*10*, *63*), the next challenge is to identify classes of problems that can be efficiently solved with algorithms that use the single-nbit and multi-nbit gate operations (Figs. 4 and 6), such as, for example, a single-nbit Deutsch algorithm shown in fig. S7. The mathematical parallels between nbit and qubit systems can offer helpful guidance in this context. In the single-nbit case, the identification of director profiles with SU(2) matrices led to Eq. 4, which reflects the Hopf fibration (*64*) of the Poincaré-Bloch sphere and allowed us to interpret nbits as superpositions of +1/2 and −1/2 defects. Therefore, a particularly interesting question, from both a mathematical and a practical perspective, is whether it is possible to construct nematic analogs of the *S*^7^ Hopf fibration that encodes the entanglement of two-qubit states (*35*, *64*). While the above nbit construction focused on defect lines in uniaxial nematics, one could use biaxial and cholesteric LCs to realize more complex non-Abelian topological structures and transformation rules (*36*, *65*) that may allow for an even broader range of logic operations in the future. The computational strategies suggested here could, in principle, also be implemented using other structured fields, such as polarized light (*66*). More generally, however, the above results suggest that nbits can provide a fruitful new paradigm for exploring the computational potential of soft matter systems.

## MATERIALS AND METHODS

### Characterization of nbit defect profiles

A starting point for creation of nbit state is a reference ∣0) defect profile

$$n_{0}=(\text{cos}\;\phi /2,\text{sin}\;\phi /2,0)$$ (7)

where ϕ is the azimuthal position on the *xy*-plane. ***n***_0_ is transformed according to ***n***(***r***) = η***n***_0_η^†^ and Eq. 1 into a general nbit configuration

$$\begin{array}{c} n(\phi )=-\text{sin}\;(\frac{\phi }{2}+\gamma -\alpha )m+\\ \text{cos}\;(\frac{\phi }{2}+\gamma -\alpha )(\text{cos}\;\beta t\times m+\text{sin}\;\beta t) \end{array}$$

where ***m*** = (cos (α + π/2), sin (α + π/2),0) = ***n***(ϕ = 2α − 2γ + π) corresponds to the director orientation at a point where it lies in the *xy*-plane, and ***t*** is a defect line tangent that is in the paper always aligned along *z* axis.

### Simulation methods

Stationary solutions of Eq. 5 were found numerically by a finite difference approach on a cubic space-time mesh. In Fig. 3, a value ϵ_a_∣***E***∣^2^*L*^2^/(4*K*) = 400 is used to achieve an adiabatic response and boundary conditions of η=1 at *z* = 0 and dη/d*z* = 0 at *z* = *L* are applied.

Nematic fields in Figs. 5 (A and B), 6, and 7were obtained by numerical minimization of the free energy with density of

$$f=\frac{A}{2}Q_{ij}Q_{ji}+\frac{B}{3}Q_{ij}Q_{jk}Q_{ki}+\frac{C}{4}{\left(Q_{ij}Q_{ji}\right)}^{2}+\frac{L}{2}\frac{\partial Q_{ij}}{\partial x_{k}}\frac{\partial Q_{ij}}{\partial x_{k}}$$ (8)

where *Q* is the nematic tensor order parameter, *L* is a nematic tensorial elastic constant, and *x_k_* is the *k*th spatial coordinate. Adopting the same values of the phase parameters *A*, *B*, *C* as in (*27*), the free energy minimum is found using a gradient descent method on a rectangular grid. Only the region of the simulation box that contains defects is shown in the figures.
