# Supplementary material for: Nematic bits and universal logic gates
Source: Sci Adv. 2022 Aug 19;8(33):eabp8371. doi: 10.1126/sciadv.abp8371 (PMC9390992; doi:10.1126/sciadv.abp8371)
Supplement: Supplementary file 1 — Supplementary text Figs. S1 to S7 [file sciadv.abp8371_sm.pdf]

Science Advances
AAAS

Supplementary Materials for

Nematic bits and universal logic gates

Žiga Kos and Jörn Dunkel

Corresponding author: Jörn Dunkel, dunkel@math.mit.edu

Sci. Adv. **8**, eabp8371 (2022)
DOI: 10.1126/sciadv.abp8371

The PDF file includes:

Supplementary text
Figs. S1 to S7
Legends for movies S1 to S3

Other Supplementary Material for this manuscript includes the following:

Movies S1 to S3

Nbit solutions for nematic defects

Stationary solutions for the director field $\boldsymbol{n}$ of a nematic liquid crystal correspond to minima of the free energy with density [38]

$$f = \frac{K}{2}(\nabla \boldsymbol{n})^2 - \frac{\epsilon_{\mathrm{a}}}{2}(\boldsymbol{n}\cdot\boldsymbol{E})^2\,, \tag{1}$$

where K denotes the elastic constant of the director field deformations, ϵ_{a} is the anisotropy of the dielectric tensor, and $\boldsymbol{E}$ is the electric field. Out of equilibrium, the director field $\boldsymbol{n}$ is driven towards a free energy minimum by a 'molecular field' $\boldsymbol{h} = -\delta f/\delta \boldsymbol{n}$, yielding the relaxation dynamics

$$\Gamma \dot{\boldsymbol{n}} = \boldsymbol{h} - \lambda \boldsymbol{n} = K\nabla^2 \boldsymbol{n} - \epsilon_a (\boldsymbol{n}\cdot\boldsymbol{E})\,\boldsymbol{E} - \lambda \boldsymbol{n}, \tag{2}$$

where Γ is the rotational diffusion constant (also known as γ_1 in the literature) and λ is a Lagrange multiplier that preserves the normalization $|\boldsymbol{n}| = 1$.

The nematic director field can contain singularities in form of defect lines, often appearing in half-integer form, in which case the director field rotates by an angle of π when circumnavigating a defect line. To obtain a director field solution close to a half-integer defect line, the Laplace operator is written in cylindrical coordinates. Then, in close proximity to the defect line, the molecular field reduces in leading order to

$$\boldsymbol{h} \approx \frac{K}{r^2}\left(\frac{\partial^2 n_x}{\partial \phi^2}\hat{\mathbf{e}}_x + \frac{\partial^2 n_y}{\partial \phi^2}\hat{\mathbf{e}}_y + \frac{\partial^2 n_z}{\partial \phi^2}\hat{\mathbf{e}}_z\right), \tag{3}$$

where r is a radial distance from the defect line and ϕ is the azimuthal angle. For small enough r, the nematic is locally in equilibrium given by the condition $\boldsymbol{n}\times\boldsymbol{h} = 0$, which is solved by the nbit form of the director field [37]

$$\boldsymbol{n}(\phi) = -\sin\left(\frac{\phi}{2} + \gamma - \alpha\right)\boldsymbol{m} + \cos\left(\frac{\phi}{2} + \gamma - \alpha\right)(\cos\beta \boldsymbol{t}\times\boldsymbol{m} + \sin\beta \boldsymbol{t})\,. \tag{4}$$

Here, $\boldsymbol{t}$ is the defect line tangent which we take in the z direction, $\boldsymbol{m} = (\cos(\alpha+\pi/2), \sin(\alpha+\pi/2), 0)$, and α, β, and γ are arbitrary angles defining the nbit state; the domain of validity of this nbit solution is also discussed in Fig. S1. In our derivation, we consider straight defect lines. In principle, they can also be distorted, which would have to be considered when deriving the nbit dynamics and implementing the logic operations. As ϕ is increased from 0 to 2π, the director field traces half of a great circle on a unit sphere as shown in Fig. 2A of the Main Text. In this work, we use quaternionic rotations of a reference director field defect profile to describe a whole range of solutions given by Eq. (4) and to construct a spinor description of nbits. Angles α, β, and γ of the nbit director field construction can be related to the quaternionic rotation of the reference director field around an axis $\boldsymbol{a}$ for an angle θ (Eq. 1 in the main text) as:

$$\alpha = \mathrm{atan2}(a_z \sin\frac{\theta}{2}, \cos\frac{\theta}{2}), \tag{5}$$

$$\beta = 2\,\mathrm{acos}\sqrt{\cos^2\frac{\theta}{2} + a_z^2 \sin^2\frac{\theta}{2}}, \tag{6}$$

$$\gamma = 2\,\mathrm{atan2}(a_z \sin\frac{\theta}{2}, \cos\frac{\theta}{2}). \tag{7}$$

The nbits in the simulations of multi-nbit logic gates are manipulated by a direct rotation of the director ansatz around defect cores. In particular the α and β angles could be directly controlled by localized electric fields that quickly decay away from the defect core.

Nbit dynamical equation

The time evolution equation for nbit solutions can be derived from hydrodynamic models of nematic liquid crystals. Our derivation below is based on the Lagrangian formalism for the nematic free energy functional and the Rayleigh dissipation function D, analogous to the derivations of dynamical equations for the $\pm 1/2$ defect motions [38] and flow-alignment [59,55] in nematics.

Dissipation function

The Rayleigh dissipation function for slow director field deformations that generate only negligible velocity fields reads

$$D = \frac{\Gamma}{2}\left(\frac{\mathrm{d}\boldsymbol{n}}{\mathrm{d}t}\right)^2 . \tag{8}$$

We want to express the dissipation function in the Pauli algebra using the Pauli matrices

$$\sigma_x = \begin{pmatrix} 0 & 1 \\ 1 & 0 \end{pmatrix}, \qquad \sigma_y = \begin{pmatrix} 0 & \mathrm{i} \\ -\mathrm{i} & 0 \end{pmatrix}, \qquad \sigma_z = \begin{pmatrix} 1 & 0 \\ 0 & -1 \end{pmatrix}. \tag{9}$$

Writing the director field in the Pauli algebra as

$$\boldsymbol{n} = n_x\sigma_x + n_y\sigma_y + n_z\sigma_z,$$

the dissipation function can be expressed as

$$D = \frac{\Gamma}{4}\,\mathrm{Tr}\left(\frac{\mathrm{d}\boldsymbol{n}}{\mathrm{d}t}\frac{\mathrm{d}\boldsymbol{n}}{\mathrm{d}t}\right). \tag{10}$$

For the director field around a defect line, we take the nbit ansatz

$$\boldsymbol{n} = \eta\boldsymbol{n}_0\eta^\dagger, \tag{11}$$

where η determines the quaternionic rotation of the reference profile

$$\boldsymbol{n}_0 = \cos\frac{\phi}{2}\sigma_x + \sin\frac{\phi}{2}\sigma_y. \tag{12}$$

Using Eq. (11), the dissipation function can be rewritten as

$$D = \frac{\Gamma}{4}\,\mathrm{Tr}\left\{\frac{\mathrm{d}}{\mathrm{d}t}\left[\eta\left(\sigma_x\cos\frac{\phi}{2} + \sigma_y\sin\frac{\phi}{2}\right)\eta^\dagger\right]\frac{\mathrm{d}}{\mathrm{d}t}\left[\eta\left(\sigma_x\cos\frac{\phi}{2} + \sigma_y\sin\frac{\phi}{2}\right)\eta^\dagger\right]\right\}. \tag{13}$$

Note that η can in principle depend on both the radial distance r and the z component, but η has no angular dependence. To obtain the dynamics of η, we integrate the dissipation function over the azimuthal angle ϕ:

$$\begin{aligned} D_\phi &= \int_0^{2\pi} \mathrm{d}\phi\, D \\ &= \frac{\pi}{4}\Gamma\,\mathrm{Tr}\left(\frac{\mathrm{d}\eta}{\mathrm{d}t}\sigma_x\eta^\dagger\frac{\mathrm{d}\eta}{\mathrm{d}t}\sigma_x\eta^\dagger + 4\frac{\mathrm{d}\eta}{\mathrm{d}t}\frac{\mathrm{d}\eta^\dagger}{\mathrm{d}t} + \eta\sigma_x\frac{\mathrm{d}\eta^\dagger}{\mathrm{d}t}\eta\sigma_x\frac{\mathrm{d}\eta^\dagger}{\mathrm{d}t} + \frac{\mathrm{d}\eta}{\mathrm{d}t}\sigma_y\eta^\dagger\frac{\mathrm{d}\eta}{\mathrm{d}t}\sigma_y\eta^\dagger + \eta\sigma_y\frac{\mathrm{d}\eta^\dagger}{\mathrm{d}t}\eta\sigma_y\frac{\mathrm{d}\eta^\dagger}{\mathrm{d}t}\right), \end{aligned} \tag{14}$$

where we have used the cyclic property of the trace and the fact that $\eta\eta^\dagger = \mathbb{1}$. We will also need the derivatives of the dissipation function with respect to $\mathrm{d}\eta/\mathrm{d}t$ and $\mathrm{d}\eta^\dagger/\mathrm{d}t$:

$$\left(\frac{\partial D_\phi}{\partial\left(\mathrm{d}\eta/\mathrm{d}t\right)}\right)^\mathsf{T} = \frac{\pi}{2}\Gamma\left[2\frac{\mathrm{d}\eta^\dagger}{\mathrm{d}t} - \mathrm{Tr}\left(\eta^\dagger\frac{\mathrm{d}\eta}{\mathrm{d}t}\sigma_z\right)\sigma_z\eta^\dagger\right], \tag{15}$$

$$\left(\frac{\partial D_\phi}{\partial\left(\mathrm{d}\eta^\dagger/\mathrm{d}t\right)}\right)^\mathsf{T} = \frac{\pi}{2}\Gamma\left[2\frac{\mathrm{d}\eta}{\mathrm{d}t} - \mathrm{Tr}\left(\frac{\mathrm{d}\eta^\dagger}{\mathrm{d}t}\eta\sigma_z\right)\eta\sigma_z\right] = \left(\frac{\partial D_\phi}{\partial\left(\mathrm{d}\eta/\mathrm{d}t\right)}\right)^*, \tag{16}$$

where we used the identity

$$\left(\frac{\mathrm{d}\eta}{\mathrm{d}t}\right)^{\dagger} = \frac{\mathrm{d}\eta^{\dagger}}{\mathrm{d}t} \tag{17}$$

and the fact that, for an arbitrary Pauli vector $\boldsymbol{p} = p_x\sigma_x + p_y\sigma_y + p_z\sigma_z$,

$$\sigma_x\boldsymbol{p}\sigma_x + \sigma_y\boldsymbol{p}\sigma_y = -\operatorname{Tr}\left(\boldsymbol{p}\sigma_z\right)\sigma_z. \tag{18}$$

Furthermore, one finds from Eqs. (15) and (16) that

$$\frac{\mathrm{d}\eta}{\mathrm{d}t} = \frac{1}{\pi\Gamma}\left\{\left(\frac{\partial D_\phi}{\partial\left(\mathrm{d}\eta^{\dagger}/\mathrm{d}t\right)}\right)^{\mathsf{T}} - \frac{1}{4}\operatorname{Tr}\left[\left(\frac{\partial D_\phi}{\partial\left(\mathrm{d}\eta^{\dagger}/\mathrm{d}t\right)}\right)^{\mathsf{T}}\sigma_z\eta^{\dagger}\right]\eta\sigma_z\right\}. \tag{19}$$

The derivation of Eq. (19) uses the identity

$$\operatorname{Tr}\left[\left(\frac{\partial D_\phi}{\partial\left(\mathrm{d}\eta^{\dagger}/\mathrm{d}t\right)}\right)^{\mathsf{T}}\sigma_z\eta^{\dagger}\right] = -\operatorname{Tr}\left[\left(\frac{\partial D_\phi}{\partial\left(\mathrm{d}\eta/\mathrm{d}t\right)}\right)^{\mathsf{T}}\eta\sigma_z\right]$$

which follows from Eqs. (15) and (16).

Elastic free energy

The elastic free energy in Eq. (1) can be decomposed into derivatives w.r.t. r, ϕ, and z. Derivatives w.r.t. ϕ describe the elastic penalty for a defect director field compared to a homogeneous director field and do not affect the nbit dynamics. Terms including the z derivatives in the elastic free energy contribution $f_{\mathrm{el}}^{z} \propto \left(\partial\boldsymbol{n}/\partial z\right)^2$ can be written as

$$f_{\mathrm{el}}^{z} = \frac{K}{4}\operatorname{Tr}\left(\frac{\partial\boldsymbol{n}}{\partial z}\frac{\partial\boldsymbol{n}}{\partial z}\right). \tag{20}$$

Following the same procedure as for Eq. (10), f_{el}^{z} can be integrated over ϕ and expressed as

$$f_{\mathrm{el}\phi}^{z} = \frac{\pi}{4}K\operatorname{Tr}\left(\frac{\partial\eta}{\partial z}\sigma_x\eta^{\dagger}\frac{\partial\eta}{\partial z}\sigma_x\eta^{\dagger} + 4\frac{\partial\eta}{\partial z}\frac{\partial\eta^{\dagger}}{\partial z} + \eta\sigma_x\frac{\partial\eta^{\dagger}}{\partial z}\eta\sigma_x\frac{\partial\eta^{\dagger}}{\partial z} + \frac{\partial\eta}{\partial z}\sigma_y\eta^{\dagger}\frac{\partial\eta}{\partial z}\sigma_y\eta^{\dagger} + \eta\sigma_y\frac{\partial\eta^{\dagger}}{\partial z}\eta\sigma_y\frac{\partial\eta^{\dagger}}{\partial z}\right) \tag{21}$$

We will further need

$$\left(\frac{\partial f_{\mathrm{el}\phi}^{z}}{\partial\left(\partial\eta^{\dagger}/\partial z\right)}\right)^{\mathsf{T}} = \frac{\pi}{2}K\left(2\frac{\partial\eta}{\partial z} + \eta\sigma_x\frac{\partial\eta^{\dagger}}{\partial z}\eta\sigma_x + \eta\sigma_y\frac{\partial\eta^{\dagger}}{\partial z}\eta\sigma_y\right). \tag{22}$$

Differentiating w.r.t. z, considering Eq. (18) and the fact that

$$\operatorname{Tr}\left(\frac{\partial\eta^{\dagger}}{\partial z}\frac{\partial\eta}{\partial z}\sigma_z\right) = 0,$$

we obtain

$$\frac{\mathrm{d}}{\mathrm{d}z}\left(\frac{\partial f_{\mathrm{el}\phi}^{z}}{\partial\left(\partial\eta^{\dagger}/\partial z\right)}\right)^{\mathsf{T}} = \frac{\pi}{2}K\left[2\frac{\partial^2\eta}{\partial z^2} - \operatorname{Tr}\left(\frac{\partial^2\eta^{\dagger}}{\partial z^2}\eta\sigma_z\right)\eta\sigma_z - \operatorname{Tr}\left(\frac{\partial\eta^{\dagger}}{\partial z}\eta\sigma_z\right)\frac{\partial\eta}{\partial z}\sigma_z\right], \tag{23}$$

which can be rewritten in the form

$$\frac{\mathrm{d}}{\mathrm{d}z}\left(\frac{\partial f_{\mathrm{el}\phi}^{z}}{\partial\left(\partial\eta^{\dagger}/\partial z\right)}\right)^{\mathsf{T}} - \frac{1}{4}\operatorname{Tr}\left[\frac{\mathrm{d}}{\mathrm{d}z}\left(\frac{\partial f_{\mathrm{el}\phi}^{z}}{\partial\left(\partial\eta^{\dagger}/\partial z\right)}\right)^{\mathsf{T}}\sigma_z\eta^{\dagger}\right]\eta\sigma_z = \pi K\frac{\partial^2\eta}{\partial z^2} - \frac{\pi K}{2}\operatorname{Tr}\left(\frac{\partial\eta^{\dagger}}{\partial z}\eta\sigma_z\right)\frac{\partial\eta}{\partial z}\sigma_z. \tag{24}$$

The same derivation can be repeated for the radial nbit dependence, yielding

$$\frac{\mathrm{d}}{\mathrm{d}r}\left(\frac{\partial f_{\mathrm{el}\phi}^{z}}{\partial\left(\partial\eta^{\dagger}/\partial r\right)}\right)^{\mathsf{T}} - \frac{1}{4}\operatorname{Tr}\left[\frac{\mathrm{d}}{\mathrm{d}r}\left(\frac{\partial f_{\mathrm{el}\phi}^{z}}{\partial\left(\partial\eta^{\dagger}/\partial r\right)}\right)^{\mathsf{T}}\sigma_z\eta^{\dagger}\right]\eta\sigma_z = \pi K\frac{\partial^2\eta}{\partial r^2} - \frac{\pi K}{2}\operatorname{Tr}\left(\frac{\partial\eta^{\dagger}}{\partial r}\eta\sigma_z\right)\frac{\partial\eta}{\partial r}\sigma_z. \tag{25}$$

Electric field

The electric field contribution to the free energy in Eq. (1) can be written as

$$f_E = -\frac{\epsilon_{\rm a}}{8}\left[\mathrm{Tr}\left(\boldsymbol{n}\boldsymbol{E}\right)\right]^2 = -\frac{\epsilon_{\rm a}}{8}\left[\mathrm{Tr}\left(\eta \boldsymbol{n}_0 \eta^\dagger \boldsymbol{E}\right)\right]^2, \tag{26}$$

where the dielectric anisotropy $\epsilon_{\rm a}$ is defined by $\epsilon_{\rm a} = \varepsilon_{\rm a}\varepsilon_0$ [38]. In close proximity to the defect line, the director field remains well described by the nbit ansatz [Eq. (4)]; however, different nbit states can have different free energy due to the electric field contribution. Using the reference profile from Eq. (12) and integrating over azimuthal angle ϕ, we obtain

$$f_{E\phi} = -\frac{\pi}{8}\epsilon_{\rm a}\left\{\left[\mathrm{Tr}\left(\eta\sigma_x\eta^\dagger\boldsymbol{E}\right)\right]^2 + \left[\mathrm{Tr}\left(\eta\sigma_y\eta^\dagger\boldsymbol{E}\right)\right]^2\right\} \tag{27}$$

$$= -\frac{\pi}{8}\epsilon_{\rm a}\left\{|\boldsymbol{E}|^2 - \left[\mathrm{Tr}\left(\eta\sigma_z\eta^\dagger\boldsymbol{E}\right)\right]^2\right\}. \tag{28}$$

The derivative of the electric free energy density w.r.t. $\eta^\dagger$ is given by

$$\left(\frac{\partial f_{E\phi}}{\partial \eta^\dagger}\right)^{\mathsf{T}} = \frac{\pi}{4}\epsilon_{\rm a}\,\mathrm{Tr}\left(\eta\sigma_z\eta^\dagger\boldsymbol{E}\right)\boldsymbol{E}\eta\sigma_z. \tag{29}$$

Final form of the dynamical equation

The Euler-Lagrange equation for the nbit dynamics reads

$$\frac{\partial D_\phi}{\partial\left(\mathrm{d}\eta^\dagger/\mathrm{d}t\right)} + \frac{\delta f_\phi}{\delta\eta^\dagger} + \lambda\eta^{\mathsf{T}} = 0, \tag{30}$$

where the Lagrange multiplier λ preserves the SU(2) structure of η and

$$\frac{\delta f_\phi}{\delta\eta^\dagger} = \frac{\partial f_\phi}{\partial\eta^\dagger} - \frac{\mathrm{d}}{\mathrm{d}r}\frac{\partial f_\phi}{\partial\left(\partial\eta^\dagger/\partial r\right)} - \frac{\mathrm{d}}{\mathrm{d}z}\frac{\partial f_\phi}{\partial\left(\partial\eta^\dagger/\partial z\right)}. \tag{31}$$

Inserting Eqs. (19), (24), (25), and (29) into Eq. (30), we obtain

$$\begin{aligned}\frac{\mathrm{d}\eta}{\mathrm{d}t} = {}& \frac{K}{\Gamma}\left(\frac{\partial^2\eta}{\partial r^2} + \frac{\partial^2\eta}{\partial z^2}\right) - \frac{K}{2\Gamma}\mathrm{Tr}\left(\frac{\partial\eta^\dagger}{\partial z}\eta\sigma_z\right)\frac{\partial\eta}{\partial z}\sigma_z - \frac{K}{2\Gamma}\mathrm{Tr}\left(\frac{\partial\eta^\dagger}{\partial z}\eta\sigma_z\right)\frac{\partial\eta}{\partial z}\sigma_z \\ & - \frac{\epsilon_{\rm a}}{4\Gamma}\mathrm{Tr}\left(\boldsymbol{E}\eta\sigma_z\eta^\dagger\right)\boldsymbol{E}\eta\sigma_z - \frac{\lambda}{\pi\Gamma}\eta,\end{aligned} \tag{32}$$

The Lagrange multiplier

$$\lambda = \frac{1}{2}\mathrm{Tr}\left[\left(\frac{\delta f_\phi}{\delta\eta^\dagger}\right)^{\mathsf{T}}\eta^\dagger\right] \tag{33}$$

ensures that

$$\mathrm{Tr}\left[\left(\frac{\mathrm{d}\eta}{\mathrm{d}t}\right)\eta^\dagger\right] = 0$$

and can be explicitly written as

$$\lambda = \frac{1}{2}\mathrm{Tr}\left\{\frac{K}{\Gamma}\left(\frac{\partial^2\eta}{\partial r^2} + \frac{\partial^2\eta}{\partial z^2}\right)\eta^\dagger - \frac{K}{2\Gamma}\mathrm{Tr}\left(\frac{\partial\eta^\dagger}{\partial z}\eta\sigma_z\right)\frac{\partial\eta}{\partial z}\sigma_z\eta^\dagger - \frac{K}{2\Gamma}\mathrm{Tr}\left(\frac{\partial\eta^\dagger}{\partial z}\eta\sigma_z\right)\frac{\partial\eta}{\partial z}\sigma_z\eta^\dagger - \frac{\epsilon_{\rm a}}{4\Gamma}\mathrm{Tr}\left(\boldsymbol{E}\eta\sigma_z\eta^\dagger\right)\boldsymbol{E}\eta\sigma_z\eta^\dagger\right\}. \tag{34}$$

Equation (32) governs the time dynamics of an nbit, with each term having an obvious physical meaning: The first term describes the elastic response due to nbit gradients in the radial and vertical direction. The expression $\eta\sigma_z\eta^\dagger$ appearing in the electric field term of Eq. (32) is equal the director normal vector $\boldsymbol{\Omega}$ that describes the nbit orientation on a unit sphere. The term $\mathrm{Tr}\left(\boldsymbol{E}\eta\sigma_z\eta^\dagger\right)$ is proportional to $\boldsymbol{\Omega}\cdot\boldsymbol{E}$. After the Lagrange multiplier is applied, the term

$\boldsymbol{E}\eta\sigma_z\eta^\dagger$ is proportional to $\boldsymbol{\Omega}\times\boldsymbol{E}$, leading to $\frac{\mathrm{d}\eta}{\mathrm{d}t}\sim(\boldsymbol{\Omega}\cdot\boldsymbol{E})(\boldsymbol{\Omega}\times\boldsymbol{E})\eta$. For the case $\epsilon_\mathrm{a}<0$ considered in this paper, the electric field aims to align $\boldsymbol{\Omega}$ along $\boldsymbol{E}$ with the speed of alignment proportional to $|\epsilon_\mathrm{a}\sin(2\xi)|$, where ξ is the angle between $\boldsymbol{\Omega}$ and $\boldsymbol{E}$.

The term

$$-\frac{K}{2\Gamma}\,\mathrm{Tr}\left(\frac{\partial\eta^\dagger}{\partial z}\eta\sigma_z\right)\frac{\partial\eta}{\partial z}\sigma_z \tag{35}$$

can be interpreted by writing the nbit derivative w.r.t. z as $\partial\eta/\partial z=\mathrm{i}\boldsymbol{a}\eta$, where $\boldsymbol{a}$ is a non-normalised vector around which the local rotation of the nbit is performed. We obtain

$$-\frac{K}{2\Gamma}\,\mathrm{Tr}\left(\frac{\partial\eta^\dagger}{\partial z}\eta\sigma_z\right)\frac{\partial\eta}{\partial z}\sigma_z=-\frac{K}{2\Gamma}\mathrm{Tr}\left(\boldsymbol{a}\eta\sigma_z\eta^\dagger\right)\boldsymbol{a}\eta\sigma_z$$

which has the same structure as the electric field term in the second line of (32). However, for $\epsilon_\mathrm{a}<0$ the sign of the term (35) is opposite to the sign of the electric field term and thus aims to align $\boldsymbol{\Omega}$ perpendicular to the rotation vector $\boldsymbol{a}$. The same interpretation can be made for the associated term with the radial derivative.

The discussion in the Main Text focusses on the regime of relatively strong electric fields with

$$\frac{\epsilon_\mathrm{a}|\boldsymbol{E}|^2L^2}{K}\gg1,$$

where L is the system size; in this case, the term (35) becomes dominated by the electric field term in Eq. (32). Indeed, for the parameters in the Main Text, our numerical simulation of Eq. (32) confirm that the term (35) can be neglected. Therefore, radially constant single-nbit solutions are governed by

$$\frac{\mathrm{d}\eta}{\mathrm{d}t}=\frac{K}{\Gamma}\frac{\partial^2\eta}{\partial z^2}-\frac{\epsilon_\mathrm{a}}{4\Gamma}\,\mathrm{Tr}\left(\boldsymbol{E}\eta\sigma_z\eta^\dagger\right)\boldsymbol{E}\eta\sigma_z-\tilde{\lambda}\eta,\qquad\qquad \tilde{\lambda}=\frac{\lambda}{\pi\Gamma} \tag{36}$$

which corresponds to Eq. (5) of the Main Text (with tildes dropped).

The dynamics of other observables, such as $\boldsymbol{\Omega}=\eta\sigma_z\eta^\dagger$, follows from Eq. (32). Finally, we also note that the nbit dynamics can be also generalized to include effects of weak anisotropy of elastic deformation modes, weak chirality, magnetic fields, and defect line curvature.

Energetic costs of nbit manipulation

In this section, we calculate the dissipated energy as a $|0)$ nbit is transformed into a $|1)$ nbit. Initial configuration of the $|0)$ nbit is a $+1/2$ nematic defect aligned along the x-axis

$$\boldsymbol{n}=(\cos\frac{\phi}{2},\sin\frac{\phi}{2},0), \tag{37}$$

where ϕ is the azimuthal angle. We perform the transformation by rotating the director field locally around the y-axis by an angle α, obtaining

$$\frac{\mathrm{d}\boldsymbol{n}}{\mathrm{d}t}=-\frac{\mathrm{d}\alpha}{\mathrm{d}t}\cos\frac{\phi}{2}(\sin\alpha,0,\cos\alpha), \tag{38}$$

The dissipated energy $\mathcal{E}$ per defect line segment L equals

$$\frac{\mathcal{E}}{L}=\int_{R_\mathrm{min}}^{R_\mathrm{max}}r\mathrm{d}r\int_0^{2\pi}\mathrm{d}\phi\int_0^\tau\mathrm{d}t\,D \tag{39}$$

$$=\int_{R_\mathrm{min}}^{R_\mathrm{max}}r\mathrm{d}r\int_0^{2\pi}\mathrm{d}\phi\int_0^\tau\mathrm{d}t\,\frac{\Gamma}{2}\left(\frac{\mathrm{d}\boldsymbol{n}}{\mathrm{d}t}\right)^2 \tag{40}$$

$$=\int_{R_\mathrm{min}}^{R_\mathrm{max}}r\mathrm{d}r\int_0^{2\pi}\mathrm{d}\phi\int_0^\tau\mathrm{d}t\,\frac{\Gamma}{2}\left(\frac{\mathrm{d}\alpha}{\mathrm{d}t}\right)^2\cos^2\frac{\phi}{2}, \tag{41}$$

where R_min and R_max are the radial bounds of the defect region, τ is the time of the transformation, and D is the dissipation function from Eq. (8). We take a constant rate of director rotation with $\frac{\mathrm{d}\alpha}{\mathrm{d}t}=\frac{\pi}{\tau}$. Also, we take $R_\mathrm{min}\to0$ and use the notation $R_\mathrm{max}=R$. The final result for the dissipated energy equals

$$\mathcal{E}=\frac{\pi^3\Gamma}{4\tau}LR^2. \tag{42}$$

Nematic Deutsch algorithm

Deutsch algorithms [60,7] played a conceptually important role in the development of quantum computation by demonstrating that certain problems can be solved exponentially faster than with classical digital computation. Broadly, Deutsch algorithms aim to determine global properties of Boolean functions $f : \{0,1\}^n \to \{0,1\}$ by using the smallest number of queries. The full entanglement-assisted power of quantum Deutsch algorithms comes into play for $n > 2$ [7], and exponential speed-ups should not necessarily be expected in nematic systems. Nonetheless, considering the elementary case $n = 1$ is useful to illustrate the differences between nbit-computations and classical digital computations. The specific goal is to use a single query to determine whether an unknown Boolean function $f : \{0,1\} \to \{0,1\}$ is balanced, $f(0) \neq f(1)$, or constant, $f(0) = f(1)$. Building on the nbit representations and nematic logic gates, we consider the logic circuit in Fig. S7, which presents the nematic analog of the quantum Deutsch algorithm in Fig. 3(b) of Ref. [7]. The computation starts from the initial two-nbit product state $|\eta_1) \otimes |\eta_2) = |0) \otimes [|0) - |1)]/\sqrt{2}$. In the first step, the phase-shifted Hadamard gate (Figs. 4D and S7) is applied to the first nbit $|\eta_1) = |0)$ to produce the superposition $|\eta_1') = [|0) + |1)]/\sqrt{2}$. As in the quantum case [7], it is assumed that the unknown Boolean black box function f acts as a 'f-controlled-NOT' defined by $|x) \otimes |y) \xrightarrow{f} |x) \otimes |y \oplus f(x))$ for $x, y \in \{0,1\}$, where '$\oplus$' represents addition modulo 2. Applying the defining [7] relations for 'f-controlled-NOT' to the product states $|0) \otimes [|0) - |1)]$ and $|1) \otimes [|0) - |1)]$ gives

$$|0) \otimes [|0 \oplus f(0)) - |1 \oplus f(0))] = (-1)^{f(0)}|0) \otimes [|0) - |1)],$$
$$|1) \otimes [|0 \oplus f(1)) - |1 \oplus f(1))] = (-1)^{f(1)}|1) \otimes [|0) - |1)].$$

Omitting the normalization factor 1/2, 'f-controlled-NOT' thus transforms the pre-black box state $[|0)+|1)] \otimes [|0)-|1)]$ into the post-black box state

$$[(-1)^{f(0)}|0) + (-1)^{f(1)}|1)] \otimes [|0) - |1)]. \tag{43}$$

The auxiliary second nbit remains unchanged throughout. Finally, applying a second phase-shifted Hadamard gate to the first nbit, the algorithm returns for the first nbit

$$(-1)^{f(0)}|f(0) \oplus f(1)) \tag{44}$$

corresponding to a $+1/2$ defect state $\pm|0)$ when f is constant, or a $-1/2$ defect state $\pm|1)$ when f is balanced (Fig. S7). Thus, by exploiting single-nbit superposition, the nematic Deutsch algorithm can determine a global property of the black box function from a single run. Note that, for the specified initial state, all operations involved only two-nbit product states and hence can be implemented using the concepts developed above. To summarize, although many quantum algorithms are unlikely to permit nematic counterparts of comparable complexity, the example in Fig. S7 suggests that suitably posed problems can be solved leveraging nbit superpositions.

Supplementary figures

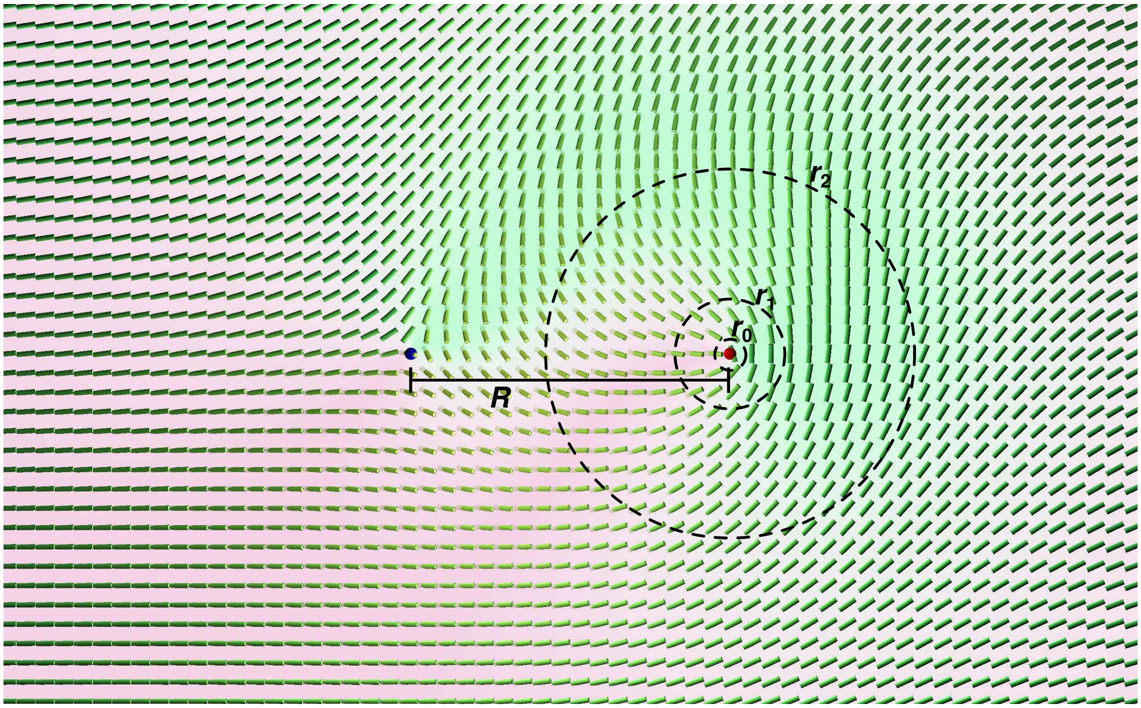

Fig. S1: Non-equilibrium director field around two half-integer nematic defect lines. The nbit solution (4) is valid in close proximity to the defect ($r_0 \ll R$). The director profile deviates from the nbit profile as one moves radially away from the defect line; however, at small distances from the defect line with respect to R ($r \sim r_1$), the director field is still close to an nbit form. By contrast, at larger distances ($r \sim r_2$), the director field profile has to be computed from the full complete time-dependent director field dynamics [Eq. (2)].

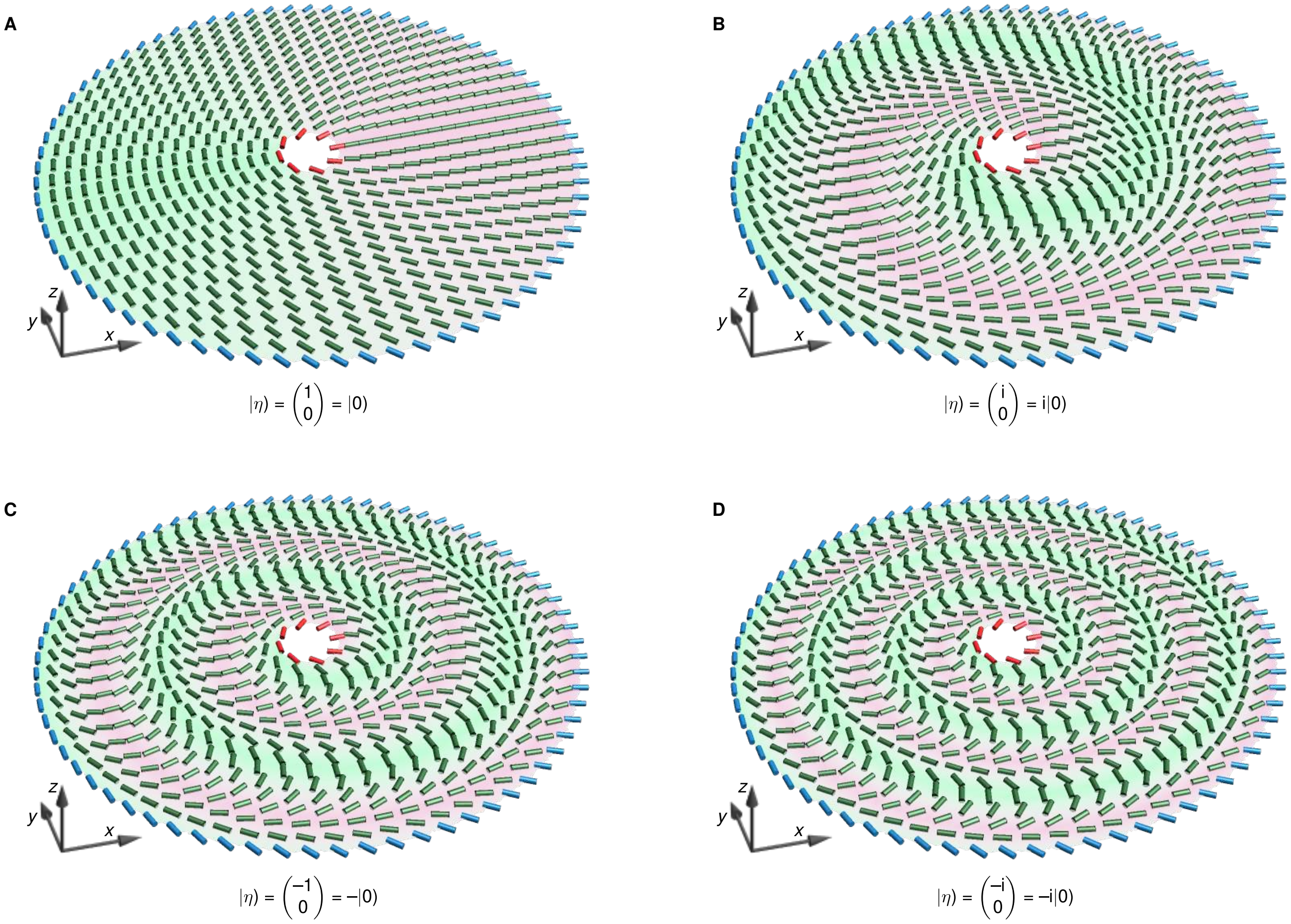

Fig. S2: Four locally equivalent configurations of the $|0\rangle$ nbit profile. The configurations are obtained by rotating the inner director field (red rods) around the z-axis while the reference director field (outer blue rods) remains fixed. For all four solutions, the inner red director field is the same, but the intermediate director field between red and blue regions is distinctively different and cannot be smoothly transformed from one configuration to another provided that inner and outer director field are kept fixed. The degree of rotation of the inner red director field equals (A) 0, (B) π, (C) 2π, and (D) 3π. A 4π-rotation can be smoothly transformed into $|0\rangle$ nbit profile, as demonstrated in Movie S2. Possible strategies to control the director field in experiment are discussed in the main manuscript.

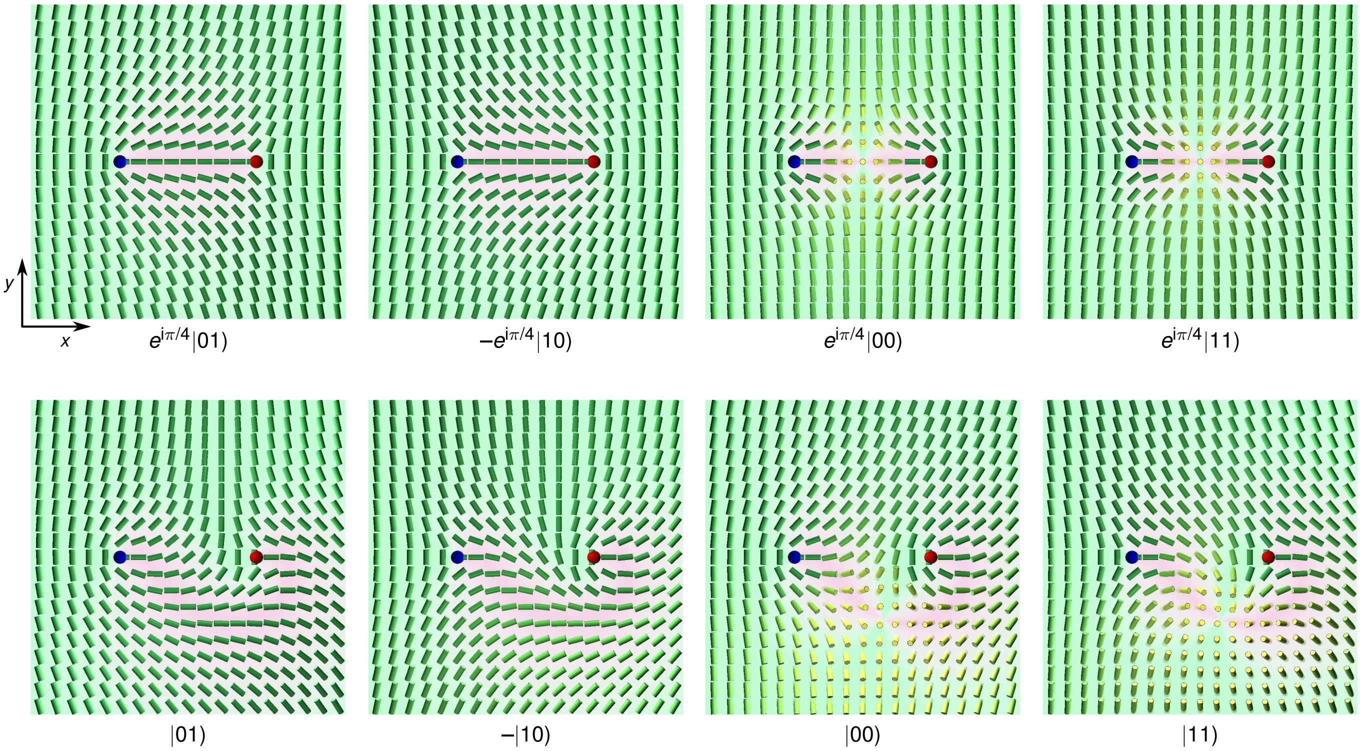

Fig. S3: Four product basis states with a global phase (top row) and without a global phase (bottom row), embedded in a director far field along y direction. Only the states $e^{i\pi/4}|01)$ and $-e^{i\pi/4}|10)$ correspond to minima of the free energy. See also Fig. S5.

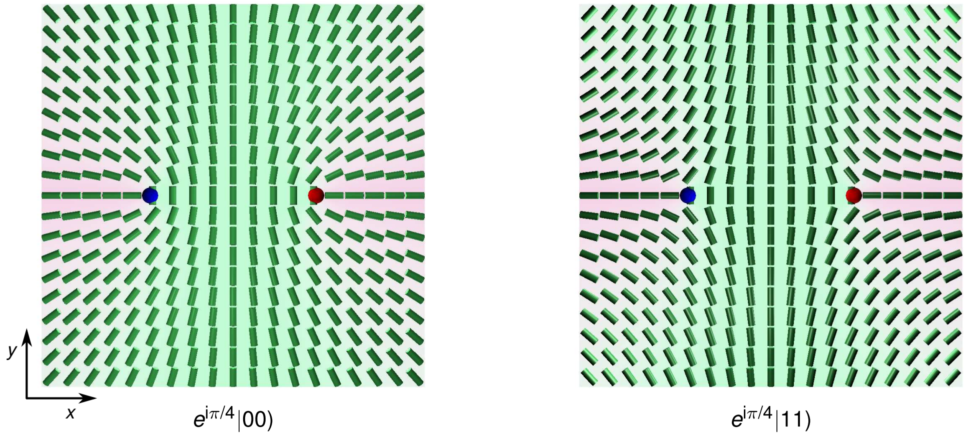

Fig. S4: Alternative realization of the two-nbit states $e^{i\pi/4}|00)$ and $e^{i\pi/4}|11)$ with the same notation but a different director structure without an umbilic soliton present. Compared to Fig. S3, the umbilic has been moved towards infinity along the y axis.

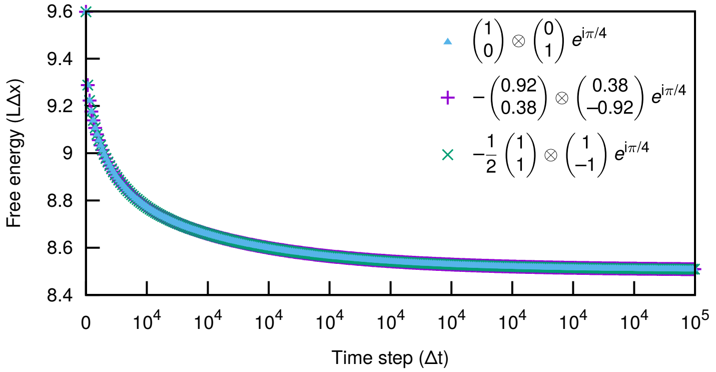

Fig. S5: Energetic equivalence of two-nbit states in the Ψ^- ensemble. We show the free energy relaxation for 3 states from the Ψ^- ensemble manifold that are also shown in Fig. 5B in the Main Text. The initial condition in the left and right half of the simulation plane corresponds to the director ansatz [Eq. (4)] for the left and right defect, respectively. Simulation was performed for periodic boundary conditions, using gradient descent as explained in Methods. Not only have the states equal final free energy, but they also follow the same relaxation curve. The same dependency is obtained for states from the Ψ^- ensemble manifold that have a far director field in an arbitrary direction.

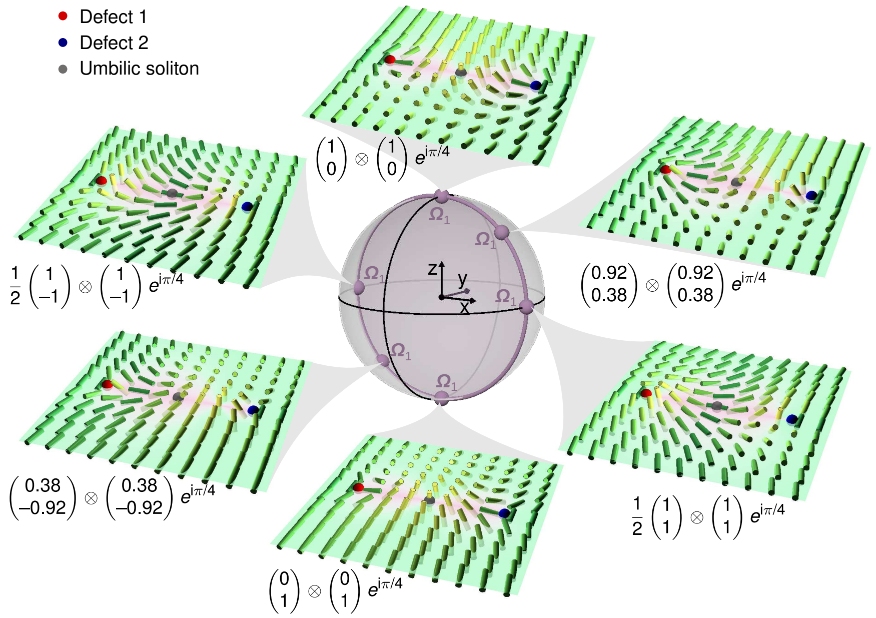

Fig. S6: Representative examples from the ensemble manifold Φ^+ of parallel two-nbit states with $\mathbf{\Omega}_1 = \mathbf{\Omega}_2$. The examples are energetically equivalent, with the director far-field parallel to the y direction. The states in the manifold are made stable by enforcing an umbilic solition at the center of the system.

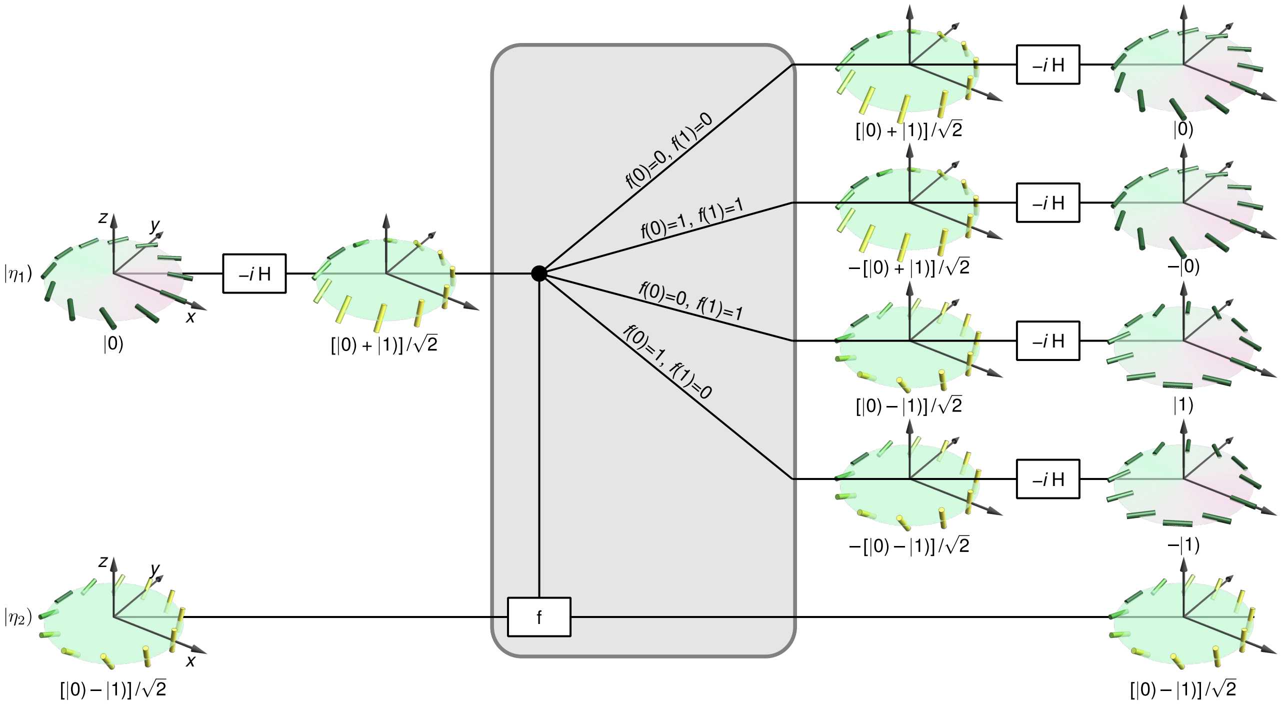

Fig. S7: Logical circuit for a single-nbit Deutsch algorithm. The goal is to determine, from a single query, whether an unknown Boolean 'black box' function $f : \{0, 1\} \rightarrow \{0, 1\}$ is constant $[f(0) = f(1)]$ or balanced $[f(0) \neq f(1)]$. The input of the algorithm is the two-nbit product state $|\eta_1) \otimes |\eta_2) = |0) \otimes [|0) - |1)]/\sqrt{2}$. The auxiliary [7] nbit $|\eta_2)$ remains unchanged throughout the computation. First, a phase-shifted Hadamard gate (Fig. 4D) $-i$H is applied to the first nbit, where the phase factor $-i$ is obtained by rotating each director around $\mathbf{\Omega}$ by an angle $-\pi$ (Fig. 2A). Next, a black box two-nbit operation of 'f-controlled-NOT' is performed on both nbits [7], changing the first nbit but not the second [Eq. (43)]. There exist four possible Boolean functions $f : \{0, 1\} \rightarrow \{0, 1\}$, each giving a different outcome for the first nbit. Upon application of a second Hadamard gate to the first nbit, the circuit will return the first nbit in a $+1/2$-defect state $\pm|0)$ if f is constant, or in a $-1/2$-defect state $\pm|1)$ if f is balanced.

Supplementary movies

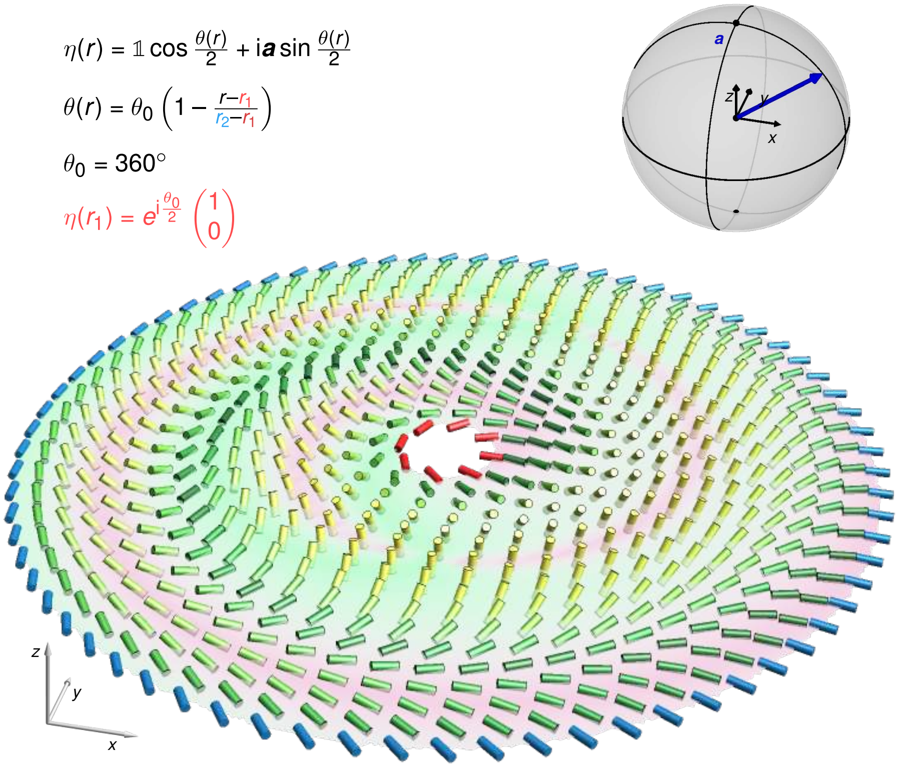

Movie S1: Creation and manipulation of the $-|0)$ nbit. During the first stage of the protocol, the $-|0)$ nbit is created by rotating the red director field (at the smallest radius shown) by 360° around z-axis, relative to the blue director field (at maximum radius) that is kept fixed in the reference configuration. The angle of rotation changes linearly with decreasing radius. Once the 360° rotation has been completed, both red and blue director fields are kept fixed. During the subsequent second stage, we change the orientation of the axis, around which the director field is rotated from the reference profile (blue) inwards. The video demonstrates how rotating the director field of a $|0)$ profile by 360° results in a $-|0)$ profile, regardless of the chosen axis of rotation. The director deformation between $|0)$ and $-|0)$ profile can therefore be smoothly transformed between clockwise spiral to anticlockwise spiral. Movie is available upon request.

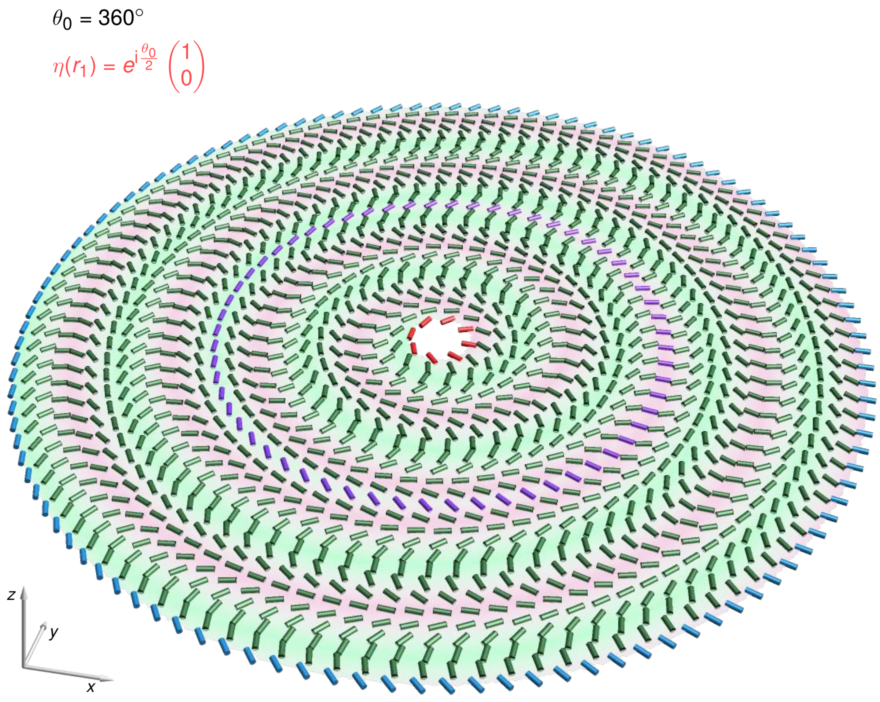

Movie S2: Nbit rotation by 720° is realized in three steps. Step 1: In the first part of the video, the inner red director field is rotated by 720° relative to the outer blue reference profile. The intermediate purple director profile gets rotated by 360° and is in the $-|0\rangle$ configuration. According to Eq. (2) in the Main Text, the red director profile should now have returned to the $|0\rangle$ configuration, since 720° rotation does not change a spinor. We show this explicitly by performing, in steps 2 and 3, a smooth reconfiguration of the director field that produces a homogeneous $+1/2$ defect director profile, while keeping the inner red and the outer blue director profiles fixed. Step 2: To achieve such a reconfiguration, we reverse the direction of the director rotation between the purple and the blue ring in the second part of the video; this process is explained in detail in Movie S1. At the end of part 2, the director field changes from clockwise to anticlockwise rotation as the radius is increased, leading to a transition from an inner right-handed to the outer left-handed spiral pattern in the phase field. Step 3: In the third part, the mismatch between clockwise and anticlockwise rotation relaxes into a homogeneous $+1/2$ director field profile. Together with Movie S1, this example shows explicitly how a rotation of the director field by 360° changes the sign of the spinor, whereas a rotation by 720° has no effect on the spinor form. Movie is available upon request.

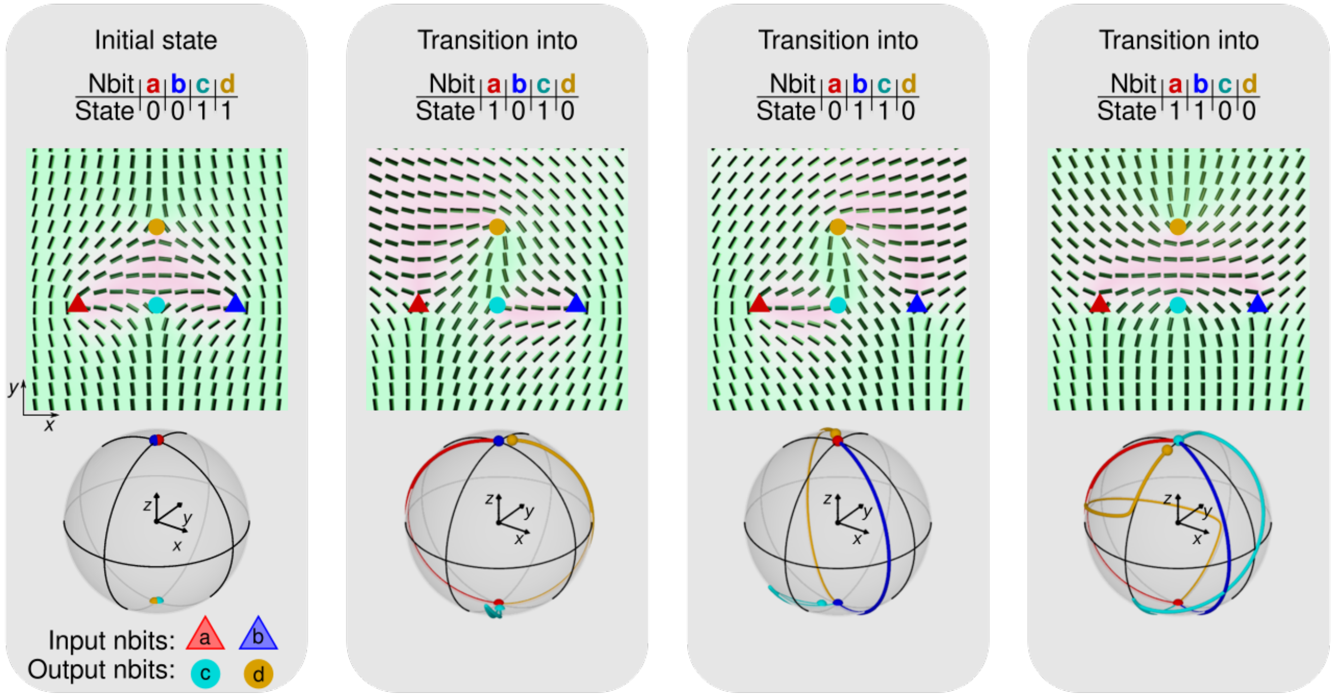

Movie S3: Universal classical logic gates. The movie shows director field reconfiguration and nbit states on the Bloch sphere corresponding to transformations in Fig. 6. First panel shows the initial state of two input nbits and two output nbits. To apply the NAND and NOR logical operation, either one or both of the input nbits 'a' and 'b' are flipped in the second, third and fourth panel, and the response of the output nbits 'c' and 'd' is observed. The movie duration is $2 \cdot 10^5 \, \Delta x^2/(\Gamma L)$, where Δx is the mesh resolution and the distance between 'a' and 'c' nbits equals $50 \, \Delta x$. Movie is available upon request.
